# Nitric Oxide-Releasing Polydimethylsiloxane Sponges with Tunable Porosity

**DOI:** 10.1021/acsami.5c06963

**Published:** 2025-06-09

**Authors:** Adam Brooks Goodman, Manjyot Kaur Chug, Natalie Crutchfield, Hitesh Handa, Elizabeth J. Brisbois

**Affiliations:** † School of Chemical, Materials, and Biomedical Engineering, College of Engineering, 1355 University of Georgia, Athens, Georgia 30602, United States; ‡ Department of Pharmaceutical and Biomedical Sciences, College of Pharmacy, University of Georgia, Athens, Georgia 30602, United States

**Keywords:** porosity, nitric oxide (NO), polydimethylsiloxane (PDMS), sponge, antibacterial

## Abstract

The popularity of sponge materials has been steadily increasing, promoting extensive research into enhancing their properties for specific applications, such as effectively separating oil from water and promoting wound healing. In this study, polydimethylsiloxane (PDMS) sponges with tunable porosity were fabricated to investigate the influence of porosity on nitric oxide (NO) donor loading, NO release kinetics, and antibacterial efficacy. The fabrication process involved a straightforward method using sodium chloride (NaCl) crystals as templates to modulate the porosity of the sponge. The PDMS sponges with varying porosities were incorporated with the NO donor *S*-nitroso-*N*-acetylpenicillamine (SNAP), using tetrahydrofuran (THF) as the embedding solvent. The results demonstrated a direct correlation between porosity and SNAP loading, diffusibility, and NO release behavior. The pore characteristics of sponges were further characterized using scanning electron microscopy. The maximum SNAP loading capacity achieved was 23 wt %, enabling substantial NO release in 4 h under physiological conditions. Moreover, the SNAP-incorporated sponges demonstrated remarkable antibacterial efficacy, with enhanced porosity correlating with higher bactericidal activity. This included a 2.45- and 2.38-log reduction in viable adhered and planktonic Escherichia coli and a 2.65- and 5.04-log reduction in viable adhered and planktonic Staphylococcus aureus compared to unmodified control PDMS sponges. These findings highlight the distinct properties exhibited by PDMS sponges with different porosities, thereby emphasizing their potential for tailoring sponge materials for specific applications such as wound healing, tissue engineering, and drug delivery.

## Graphical abstract

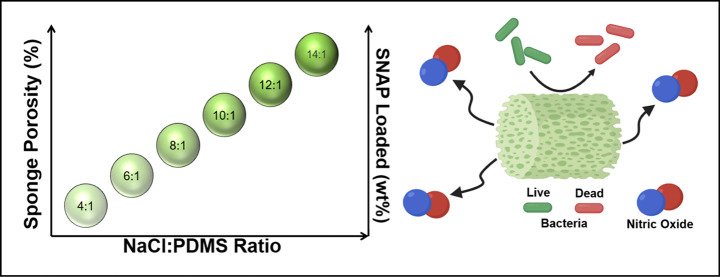

## 1 Introduction

Due to their diverse applications, sponge materials have gained significant attention in engineering and medicine. Polydimethylsiloxane (PDMS) sponges, in particular, have shown promise in various fields, as they are distinguished by their porosity, which controls properties like mechanical robustness and therapeutic molecule loading capabilities. These characteristics position PDMS sponges as an optimal selection for tissue engineering, oil–water separation, electronics, and more.^1–5^ Fabrication methods, such as freeze-drying, sugar cube templating, and salt removal, have been developed to tailor sponges with specific properties.^2,6,7^

Polydimethylsiloxane stands out as a versatile material, especially in the biomedical engineering field, due to its unique combination of inherent inertness, thermal stability, flexibility, and hydrophobicity derived from the Si–O backbone.^8^ The hydrophobic nature allows for its effective use in applications such as oil–water separation and removal of heavy metal ions from polluted water.^2,9^ Furthermore, the feasible fabrication of PDMS sponges is cost-effective and environmentally friendly, as PDMS can hydrolyze into small, water-soluble compounds under abiotic conditions, ensuring safe disposal.^10–12^

Porosity serves as a key factor in shaping multiple properties of PDMS sponges. By increasing the sponge’s porosity, mechanical robustness becomes compromised while accommodating other characteristics, such as improved cell attachment and better exchange of oxygen and nutrients, which are valuable in tissue engineering applications.^13^ While PDMS sponges have been extensively studied in some areas, the specific influence of porosity on drug loading and release kinetics remains unexplored territory.

Incorporating functional elements into PDMS sponges opens up new possibilities for advanced applications. One such agent is nitric oxide (NO), a small molecule synthesized by various cell types in the human body. At low concentrations (nM range), NO acts as a signaling molecule, regulating numerous physiological processes, while at higher concentrations (μM range), it exhibits potent antibacterial properties, disrupting formed biofilms.^14,15^ To harness the potential of NO, *S*-nitrosothiols (RSNOs), like *S*-nitroso-*N*-acetylpenicillamine (SNAP), have been used for the controlled release of NO triggered by factors including heat, light, and metal ions.^16^ Integrating NO-releasing chemistry into PDMS sponges offers exciting possibilities for numerous applications. These include introducing an antibacterial agent to minimize bacterial attachment and dispersion on medical devices, enhancing cell attachment and proliferation for tissue engineering, and preventing thrombosis to avoid unwarranted blood clots.

This study investigates the incorporation of SNAP into PDMS sponges to assess the impact of porosity on NO release. Nitric oxide-releasing materials are known for their broad-spectrum antimicrobial properties. By introducing SNAP at a concentration of 25 mg/mL, the PDMS sponge gains NO-releasing capabilities in the μM range, providing it with antibacterial properties. This positions the sponge as a highly promising candidate for wound healing, infection prevention of catheter insertion sites, and medical device decontamination.^17^ The inherent hydrophobicity of PDMS may restrict its suitability for wound healing applications. However, introducing hydrophilic modifications, such as incorporating a hydrophilic surfactant could enhance its potential for these uses. Regardless, this type of material allows for enhanced NO-donor loading potential, capitalizing on the porous structure of the PDMS-based sponge. While previous studies have explored NO-releasing materials across applications such as extracorporeal circuits and indwelling catheters,^18–24^ the distinctiveness of this study lies in the material’s porosity. This unique feature facilitates NO-donor loading into the material, thereby enhancing bactericidal activity against adhered and planktonic bacteria. This work aims to demonstrate that by leveraging porosity, significant reductions in bacterial adhesion and growth can be achieved, particularly targeting E. coli and S. aureus. Such integration holds substantial potential for advancing the field of biomedical engineering. Sponges loaded with SNAP were fabricated using a facile salt template removal technique.^2^ By employing tetrahydrofuran (THF) as the SNAP-carrier agent, SNAP was incorporated into the polymer matrix of PDMS sponges with varying porosity (60–89%). The characterization of the sponges included assessing their SNAP loading and NO release activity, along with using scanning electron microscopy techniques to gain insight into the porosity of the different sponge structures. Subsequently, the diffusibility of SNAP from the sponges in solution, and their *in vitro* antibacterial efficacy, was evaluated. This technology holds great promise in biomedical applications, offering a versatile approach to combat infections, promote tissue regeneration, and enhance various medical procedures.

## 2 Materials and Methods

### 2.1 Materials

Poly­(dimethylsiloxane) (PDMS) base agent (Sylgard 184A) and thermal curing agent (Sylgard 184B) were bought from Ellsworth Adhesives. Crystalline sodium chloride, concentrated hydrochloric acid (conc. HCl, 12.1 M), and ethanol (100%) were purchased from Fisher Scientific, Inc. Luria–Bertani (LB) broth, LB agar, phosphate buffered saline (PBS), sodium nitrite (>99.0%), methanol (>99.8%), concentrated sulfuric acid (conc. H_2_SO_4_, 18 M), ethylenediaminetetraacetic acid (EDTA), *N*-acetylpenicillamine (NAP), and tetrahydrofuran (THF) were purchased from Sigma-Aldrich (St. Louis, MO). Phosphate buffered saline (10 mM, pH 7.4) and LB media were dissolved in water and sterilized in an autoclave at 121 °C, 100 kPa (15 psi) above atmospheric pressure for 30 min prior to bacterial studies. Milli-Q filter was used to obtain deionized (DI) water for preparation of the aqueous solutions. Escherichia coli (ATCC 25922) and Staphylococcus aureus (ATCC 6538) were obtained from the American Type Culture Collection (Manassas, VA).

### 2.2 Material Synthesis

#### 2.2.1 SNAP Synthesis and Purity

Synthesis of SNAP was carried out using a slightly modified version of a previously reported method, resulting in crystals with a purity exceeding 90%.^24^ Purity was confirmed with the ^1^H NMR spectra of SNAP used in the sponges (Figure S1).^25^ Briefly, 5 g sodium nitrite was dissolved in 40 mL DI water then added dropwise to a stirring solution containing 5 g NAP in 60 mL methanol, 5 mL concentrated H_2_SO_4_ and 20 mL HCl. The reaction beaker was placed into an ice bucket and continuously purged with nitrogen for 8 h without stirring. Crystals were collected via vacuum filtration, gently washed with 100 μL chilled DI water, and placed into a desiccator to dry overnight. The solution and crystals were protected from light during each step. Purity of the crystals was also measured using a chemiluminescence nitric oxide analyzer (NOA 280i, Sievers, Boulder, CO) by quantifying the moles of NO released from a known concentration of SNAP with 50 mM copper chloride and 10 mM cysteine.

#### 2.2.2 Fabrication of PDMS Sponges

Sponge formulation methods were kept consistent with the amount of sodium chloride (NaCl) incorporated ranging from 16 to 64 g. Thorough mixing of PDMS base (4 g) and curing agent (0.4 g) was employed for 2 min. Subsequently, NaCl crystals were manually stirred in for 2 min until combined, resulting in a wet, sand-like mixture. This mixture was compacted into cylindrical glass molds (3.8 cm inner diameter, 1.3 cm height) and cured at 100 °C for 15 h. Once cured, the PDMS sponges with varying salt concentrations were removed from the oven and placed in a beaker filled with DI water maintained at 100 °C using a thermocouple for a minimum of 8 h to dissolve the salt template. Sponges were removed from their molds, dried in an oven at 100 °C for 2 h, and washed with ethanol to remove any residual salt on the outer surface. Using a 4.5 mm diameter biopsy punch-out, dry PDMS sponges were cut out and trimmed to a thickness of 5 mm and used for all experiments unless stated otherwise.

#### 2.2.3 SNAP Incorporation into Sponges

Nitric oxide-releasing SNAP was incorporated into the PDMS sponges by immersing them in a solution of SNAP in THF (25 mg/mL). The sponges were allowed to swell in this solution for 24 h to ensure optimal absorption of SNAP throughout the sponge matrix. Samples were then dried for 24 h in a dark environment within a fume hood and stored in a −20 °C freezer until further use.

### 2.3 Sponge Characterization

#### 2.3.1 Porosity

The porosity of control PDMS sponges, referred to as their initial porogen concentration (16, 24, 32, 40, 48, 56, and 64 g), was determined through a water replacement method. As PDMS is hydrophobic, sponge samples were submerged in ethanol for 4 h for complete swelling. Swollen sponges were then placed in water that was replaced every hour for 4 h then left for 24 h to allow the water to fully replace the ethanol within the sponges. The porosity of sponge samples was then calculated using the mass per volume density measured before and after swelling using eq 1.

1 $$\mathrm{Porosity}\;\left(\%\right)=\left(1-\frac{p}{p_{s}}\right)\times 100\%$$

Where *p* is the density of the sponge and *p*_s_ is the density of the swollen sponge after immersing in distilled water for 24 h. The porosity of SNAP-incorporated sponges was not examined as ethanol quickly dissolved SNAP from the sponges.

#### 2.3.2 Scanning Electron Microscopy and Energy-Dispersive X-ray Spectroscopy

Microscopy and spectroscopy techniques were used to investigate the surface morphology, pore size, and elemental compositions of the different PDMS sponges. The samples, with an approximate diameter of 6 mm, were prepared for imaging by sputter coating with a 10 nm gold–palladium coating using a Leica sputter coater (Leica Microsystems). Field emmsion scanning electron microscopy (FEI Teneo, FEI Co.) was employed to obtain detailed images of the cross-sectional morphology and porosity of the different sponge formulations. Energy-dispersive X-ray spectroscopy (EDS) was used to detect the presence and distribution of sulfur in the SNAP-sponges. To assess the pore size distributions, Gaussian least-squares fitting was used and analyzed using ImageJ imaging software from National Institutes of Health (Bethesda, MD). Each sponge had 30 randomly selected sites analyzed for each sample formulation to obtain representative pore size distribution data.

#### 2.3.3 SNAP Loading

The extent of SNAP loaded into the sponges was assessed by measuring the concentration of SNAP in each sample using a UV–vis spectrophotometer (Cary 60, Agilent Technologies). Sponges were placed into 10 mL of THF for 1 h to fully extract the SNAP before adding 1 mL of solution to a quartz cuvette. The absorbance was measured at 340 nm where the S-NO group of SNAP emits an absorbance maxima.^26^ The molar absorptivity of SNAP in THF was determined to be 951 M^–1^ cm^–1^ at the absorbance maxima. Using a standard curve of SNAP in THF, the concentration of SNAP was determined and converted to a weight percent of the sponge.

#### 2.3.4 SNAP Diffusibility

Diffusion of SNAP from the sponges was quantified by submerging samples into 1.5 mL microcentrifuge tubes containing 1 mL of 10 mM PBS, pH 7.4, with 100 μM EDTA. The amount of SNAP diffused into the 1 mL samples was determined using a UV–vis spectrophotometer. Samples were stored in the dark at 37 °C, and after each extraction (1, 4, 8, 12, and 24 h), PBS with EDTA was replenished to the tubes. Using a standard curve of 10 mM PBS with 100 μM EDTA, the molar absorptivity of SNAP at 340 nm was observed to be 1025 M^–1^ cm^–1^. Sample measurements were normalized to the weight of the sample (mg SNAP/mg sample).

#### 2.3.5 Measuring Real-Time NO Release under Physiological Conditions

Nitric oxide release from the SNAP sponges was analyzed using a chemiluminescence NOA. In this method, NO was continuously purged from the samples and directed into an internal reaction chamber, where it reacted with ozone, forming excited nitrogen dioxide. The relaxation of this excited state released a photon, detected by a photomultiplier tube. The intensity of the photon’s light was directly proportional to the amount of NO released from the sample, allowing for precise measurements.^21,23^ The quantification of NO was expressed in parts per billion (ppb) and used to determine the NO flux by normalizing the samples to their weights (× 10^–10^ mol mg^–1^ min^–1^).

To simulate a physiologically relevant environment, the sponge samples were submerged in 37 °C PBS, pH 7.4, with 100 μM EDTA. A circulating water bath was used to maintain the sample cell temperature at 37 °C. After establishing a baseline measurement with an amber cell chamber containing 3 mL of PBS, the sponges were introduced to the chamber, and NO release was continuously monitored for 4 h.

### 2.4 Assessment of Antibacterial Properties

#### 2.4.1 Growth of Microbial Cultures

A single isolated colony of Escherichia coli (E. coli) and Staphylococcus aureus (S. aureus) was inoculated in LB broth and incubated at 37 °C for 15 h at 150 rpm. Bacterial proliferation in media overtime was monitored by measuring the optical density (OD) of the microbial suspensions at 600 nm wavelength using a UV–vis spectrophotometer. The bacterial suspensions were reinoculated, and the mid log phase was extracted by centrifuging the culture at 4400 rpm for 7 min. Bacterial OD was adjusted to 0.1 corresponding to ∼10^7^ Colony Forming Units (CFU)/mL and used for further analysis.

#### 2.4.2 Adhered Bacterial Assay

A 4 h bacterial adhesion assay was conducted to assess the antibacterial activity of the NO-releasing sponges. Control (no SNAP) and SNAP PDMS sponges were submerged in 1.5 mL microcentrifuge tubes containing 1 mL of adjusted bacterial suspension (0.1 OD, ∼10^7^ CFU/mL) in sterile 10 mM PBS. After incubating for 4 h at 37 °C on a shaker at 150 rpm, sponges were gently rinsed once in sterile 10 mM PBS to remove any unadhered bacteria from the sample. Samples were placed into 15 mL conical vials containing 1 mL of sterile 10 mM PBS where they were homogenized and vortexed for 1 min each to remove all adhered bacteria. Second and fourth dilutions of each sample were evenly spread on LB agar plates (50 μL) using a cotton applicator and incubated at 37 °C for 24 h. The grown colonies were enumerated to calculate the number of viable CFUs per mg of sponge. Percent reduction in viable bacteria compared to respective controls was calculated using eq 2. Additionally, eq 3 was employed to determine the log reduction of viable bacteria exhibited by each sponge type compared to their respective controls without SNAP.

2 $$\mathrm{Bacterial Reduction}\;\left(\%\right)=\frac{C_{\mathrm{PDMS}}-C_{\mathrm{SNAP}}}{C_{\mathrm{PDMS}}}\times 100\%$$

3 $$\log\mathrm{Reduction of Viable Bacteria}={\mathrm{Log}}_{10}\left(\frac{C_{\mathrm{PDMS}}}{C_{\mathrm{SNAP}}}\right)$$

Where *C* represents the concentration of viable bacteria in CFU mg^–1^.

#### 2.4.3 4 h Planktonic Bacterial Assay

A planktonic bacterial viability assay was conducted over 4 h to assess the antibacterial activity of the NO-releasing sponges in solution. The bacterial solution used to expose control (no SNAP) and SNAP PDMS sponges in the adhesion assay were examined for their bacterial viability. After samples were removed from the exposure inoculum, the resulting solutions were appropriately diluted and 50 μL were spread on LB agar plates using a cotton applicator and incubated at 37 °C for 24 h. The grown colonies were enumerated to calculate the number of viable CFUs per mL of solution and normalized to the weight of the sponge. Percent and log reductions in viable bacteria compared to respective controls were calculated using eqs 2 and 3, where *C* represents the concentration of viable bacteria in CFU mg^–1^ mL^–1^.

### 2.5 Statistical Analysis

Data are expressed as mean ± standard error of the mean (SEM) unless stated otherwise. All analysis calculations were conducted using Prism 9.1 (GraphPad Software, San Diego, CA). A standard one-way analysis of variance (ANOVA) was used to perform statistical comparisons among the treatment groups by evaluating the average values. Multiple comparisons were conducted to assess the differences between the sample group averages. Values of *p* < 0.05 were considered statistically significant. Two replicate experiments were conducted for all biological studies.

## 3 Results

### 3.1 Sponge Characterization

#### 3.1.1 Sponge Fabrication and Incorporation of NO Donor

Polydimethylsiloxane sponges were fabricated using crystalline NaCl as the porogen, following similar methods previously reported.^2,27^ Upon immersion in hot water, cured PDMS incorporated with NaCl underwent a dissolution process, leading to the removal of NaCl particles and the creation of void spaces. The rate of this process notably accelerated with higher porogen concentrations, promoting dissolution throughout the PDMS matrix. This, in turn, facilitated a more rapid influx of water, expediting the removal of the salt template. In contrast, sponges with lower porogen concentrations required longer dissolution periods of up to 7 days.

Ultimately, this process resulted in forming a porous network characterized by interconnected tunnels within the PDMS material, imparting sponge-like porosity. To integrate NO-releasing chemistry, SNAP was embedded throughout the PDMS matrix and pores of the sponges. Sponge samples were immersed in a SNAP-THF solution (25 mg/mL) and dried until complete THF evaporation. Sponges were observed with a green coloration, confirming the successful incorporation of SNAP into the PDMS matrix (Figure 1A).

**Figure 1:**
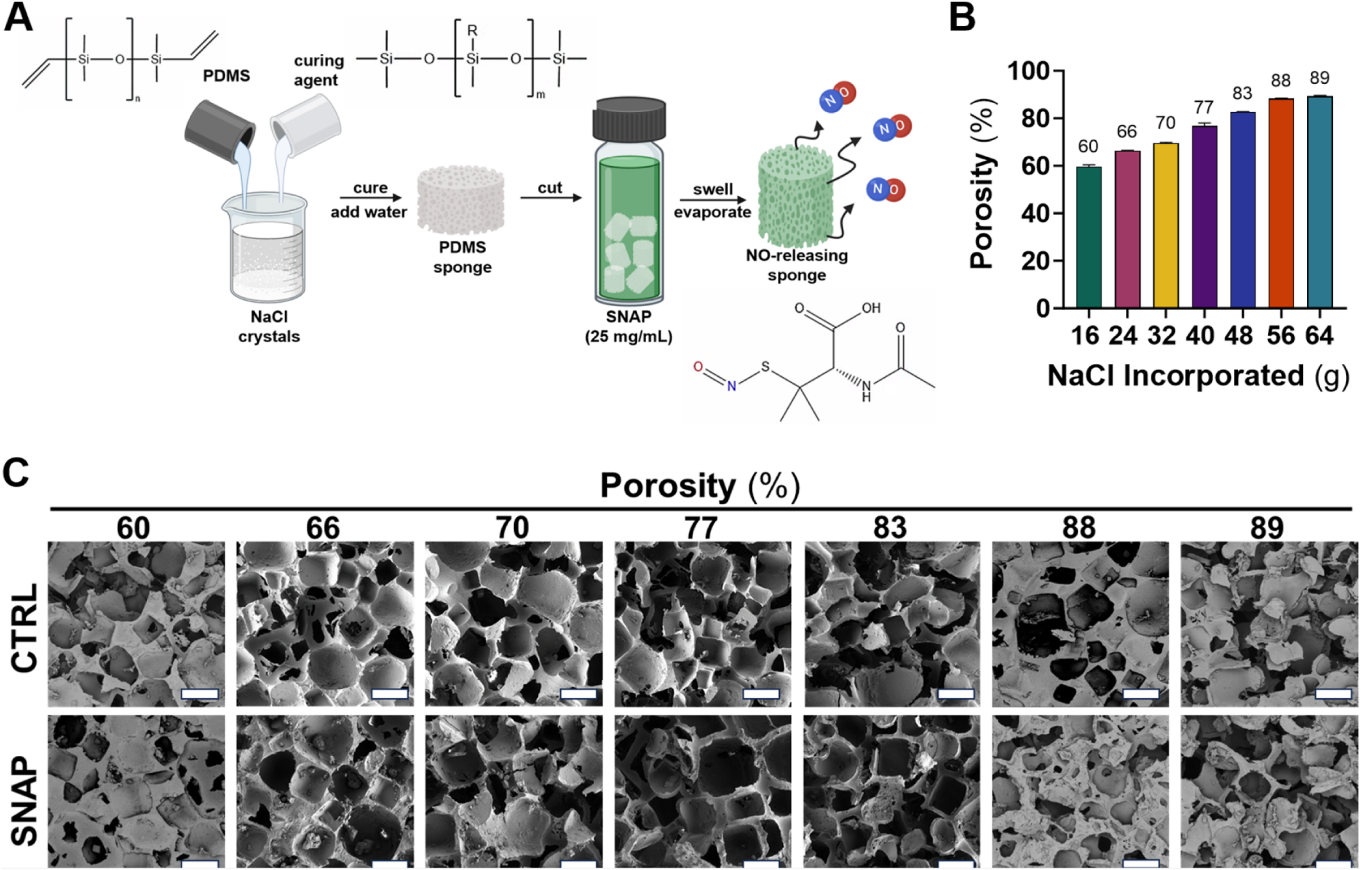
Fabrication of PDMS sponges with varying porosity. (A) Fabrication schematic of SNAP-incorporated PDMS sponges using THF as the solvent. (B) Porosity measurements demonstrated an increasing trend as more NaCl was added to the initial formulation. Average porosity values (%) for each sponge type are shown on top of corresponding bars. Data are presented as mean ± SEM (*n* = 3). (C) Respective scanning electron microscopy images of control and SNAP-incorporated PDMS sponges with scale bars corresponding to 500 μm.

#### 3.1.2 Porosity and Wettability

Controlling PDMS porosity enables the creation of a sponge-like material with distinct characteristics, such as cell attachment and mechanical integrity. The ratio of void space to PDMS polymer in each sponge type was determined by analyzing the change in density following a 24 h swelling process with water.^3^ The porosity increased almost proportionally with higher quantities of NaCl added to the initial formulation (Figure 1B). The quantity of NaCl was carefully selected in relation to the PDMS, encompassing ratios ranging from 4:1 to 16:1 of NaCl to PDMS. The corresponding porosity values exhibited a gradual rise, ranging from 60% to 89%. Each sponge type exhibited a statistically significant difference from the others, except for the 56 and 64 g sponge types, which showed no significant variation.

Following the supporting methods (Section S1.1), the hydrophobicity of the sponges was assessed to determine the impact of SNAP incorporation on the wettability. Static contact angle measurements showed no significant differences between the tested sample types (Figure S2). Additionally, porosity had no notable effect on the hydrophobic nature of PDMS, with contact angles of 114 ± 3.44° and 113 ± 4.72° observed for 60% and 89% porous PDMS sponges, respectively, consistent with previously reported values for porous PDMS materials.^28^ Furthermore, the incorporation of SNAP did not significantly alter the hydrophobic properties of the sponges.

#### 3.1.3 Scanning Electron Microscopy and Energy-Dispersive X-ray Spectroscopy

Scanning electron microscopy was used to examine the cross-sectional morphology of the sponges, providing further insight on their porosity and macroscopic network structure. The imaging analysis highlighted the porous nature of the sponges (Figure 1C). Pore size distributions were determined by analyzing 30 random sites from each sponge formulation to investigate the pore characteristics. Increasing porogen concentration in the sponge fabrication did not display a systematic trend in average pore size (Table 1), suggesting variability in porogen packing may influence final pore formation. Although the addition of SNAP to the PDMS matrix did not result in a statistically significant change in average pore size, a slight reduction was observed across all sponge types. This may be attributed to swelling-induced expansion of the polymer matrix by SNAP or potential aggregation of SNAP within the pores, which could partially obstruct or constrict the pore openings.

**Table 1:** Representative Pore Size Distribution of PDMS and SNAP-Incorporated Sponges

Sponge Porosity (%)	Control Pore Size (μm)	SNAP Pore Size (μm)
60 ± 0.86	248.2 ± 19.88	219.4 ± 13.76
66 ± 0.04	261.8 ± 17.86	234.0 ± 18.82
70 ± 0.35	254.7 ± 21.68	221.2 ± 12.26
77 ± 1.17	228.3 ± 15.61	216.4 ± 19.58
83 ± 0.13	265.3 ± 19.09	247.6 ± 12.37
88 ± 0.17	211.0 ± 14.84	195.1 ± 14.94
89 ± 0.31	207.5 ± 14.76	194.2 ± 15.52

To confirm SNAP incorporation in the sponge samples (60, 77, 89% porous), sulfur from the S-NO bond of SNAP molecules was mapped via EDS. Cross-sectional imaging detected sulfur throughout the sponges, indicating successful SNAP loading (Figure S3). Notably, higher porosity correlated with increased sulfur detection, suggesting greater SNAP incorporation. The swelling method used for SNAP loading allows for its recrystallization within the polymer matrix. However, in porous materials, SNAP may also accumulate around pore structures, making it more readily accessible in physiological environments. As porosity increases, denser regions of sulfur were observed, potentially indicating SNAP aggregation along the pores.

#### 3.1.4 Evaluation of Compressive Strength

Although no visual differences were observed in the macroscopic network of the sponges, crystallizing SNAP within the material may affect the mechanical integrity of the sponges. Following the supporting methods (Section S1.2), the compressive modulus of the 60, 77, and 89% porous sponges was calculated to assess the effect of porosity and SNAP incorporation on compressive strength. All sponge types returned to their original height immediately following compression. As porosity increased, the force required to compress the sponge from 10% to 40% strain significantly decreased due to the greater pore volume within the material (Figure S4). However, SNAP incorporation increased the force required to compress the sponges. The compressive strength of the 60% porous sponge increased from 11.6 ± 1.72 kPa to 15.4 ± 2.70 kPa after SNAP incorporation, while the 89% porous sponge increased from 0.050 ± 0.013 kPa to 0.23 ± 0.12 kPa. The compressive modulus of the PDMS sponges is consistent with values reported for other PDMS-based porous materials. For example, although fabricated using a different method, a PDMS sponge with ∼66% porosity exhibited a compressive stress of approximately 5 kPa at 30% strain.^29^ Similarly, PDMS sponges prepared using NaCl as a porogen have demonstrated a porosity-dependent trend in mechanical properties, with compressive moduli ranging from ∼0.25 kPa down to <0.1 kPa as porosity increased from ∼71% to ∼85%.^28^ The sponges in this work follow a comparable trend, where increasing porosity led to a reduction in compressive modulus. These results support the conclusion that both porosity and chemical additives like SNAP can significantly influence the mechanical behavior of PDMS sponges, which is particularly important when considering their handling and durability in biomedical applications.

#### 3.1.5 Investigation of Fluid Absorption Using Simulated Wound Medium

Following supporting methods (Section S1.3), the swelling behavior of sponges (60, 77, and 89%) was evaluated in PBS and simulated wound fluid (SWF), the latter composed of an equal volume of PBS and fetal bovine serum (FBS).^30^ Samples were incubated for 48 h, and swelling capacity was quantified using Equation S1 (Figure S5). For the 60% porous control sponges, swelling behavior was comparable in both PBS and SWF after 48 h, indicating limited fluid uptake due to low porosity. However, SNAP incorporation slightly enhanced swelling, particularly in SWF, where 60% porous SNAP sponges reached 210 ± 8.42% swelling compared to 165 ± 12.8% in PBS. The 77% porous sponges demonstrated greater overall swelling. In SWF, 77% porous control sponges reached 304 ± 43.7%, while SNAP-incorporated sponges reached 398 ± 4.77%. For 89% porous sponges, both SNAP and control groups absorbed more in SWF than in PBS. Control sponges swelled to 516 ± 55.3% in SWF and 331 ± 53.7% in PBS, whereas SNAP-incorporated sponges swelled to 507 ± 33.8% in SWF and 377 ± 32.9% in PBS. However, the differences between SNAP and control sponges at 89% porosity were minimal in both media. Given the marginal rise in porosity when shifting from a NaCl to PDMS ratio of 14:1 to 16:1, further investigation of the 89% porous sponge was deemed unnecessary.

### 3.2 NO Activity of Sponges

#### 3.2.1 SNAP Loading

The SNAP loading ability of sponges was evaluated using a swelling method, which ensured direct incorporation of SNAP into the PDMS polymer matrix (Figure 2A).^31^ It was observed that as the porosity of the sponge increased, the capacity to load SNAP also rose correspondingly (Figure 2B). Significant differences in SNAP loading were evident across all sponge types, except for the 60 and 66% porous sponges (Table S1). This may be attributed to the limited NaCl concentration used during fabrication, leading to fewer interconnected pores within the sponge and, consequently, impeding the full saturation of SNAP within the PDMS matrix.

**Figure 2:**
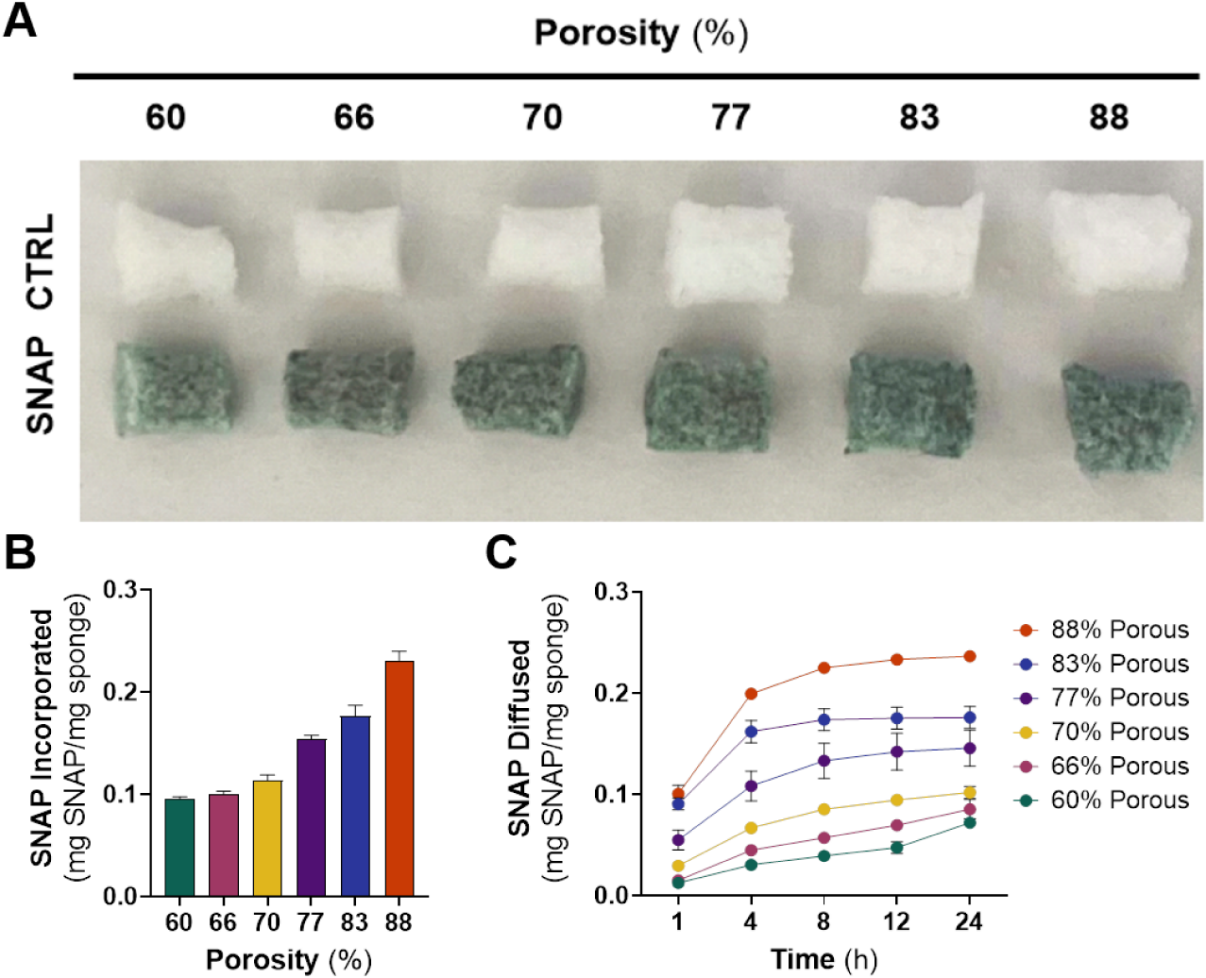
SNAP integration and diffusion of PDMS sponges. (A) Representative control and SNAP-incorporated PDMS sponges. (B) SNAP loading of sponges demonstrated the increased capacity of SNAP as porosity increases. (C) Diffusion of SNAP over 24 h highlighted a faster diffusion rate of SNAP into the PBS buffer as porosity increases. Data are presented as mean ± SEM (*n* = *3*).

Increasing the quantity of NaCl in the PDMS prepolymer leads to a higher concentration of closely packed NaCl particles within the cured structure. This results in enhanced interconnected tunnel formation when the NaCl dissolves, facilitating a more efficient dispersion of the SNAP solution throughout the PDMS matrix. As a result, SNAP is embedded both within the PDMS matrix and along the surfaces of the sponge’s pores, contributing to a higher amount of SNAP incorporated per surface area of the sponge.

This enhancement in SNAP loading highlights the clear advantage of the porous sponge structure, enabling superior drug incorporation capabilities and presenting promising opportunities for NO donor delivery applications. Moreover, the tunability of the sponge’s porosity opens up possibilities for tailoring drug delivery properties, optimizing drug release kinetics, and further enhancing therapeutic outcomes.

#### 3.2.2 SNAP Diffusibility

The diffusion of SNAP from the sponges was monitored over a 24 h period using PBS with EDTA as the extracting medium. This investigation aimed to assess how porosity influences the leaching behavior of SNAP from the sponge, while quantitatively analyzing its diffusion kinetics to evaluate its potential as a drug delivery system. Measuring SNAP diffusion in solution provides insight into the release dynamics of the NO donor, which directly influences NO availability and antibacterial efficacy. The 77–88% porous sponges exhibited SNAP diffusions of 0.15, 0.18, and 0.24 mg of SNAP per mg of sponge within 24 h, corresponding to over 90% of the total SNAP being released (Figure 2C). Notably, the 83 and 88% porous sponges exhibited exceptional diffusibility within 4 h. In a mere additional 4 h, the 77% porous sponge diffused a comparable amount of SNAP, with 13% of its total SNAP content available after 8 h. In contrast, the 60, 66, and 70% porous sponges displayed slower diffusion kinetics, diffusing 0.07, 0.09, and 0.10 mg of SNAP per mg of sponge after 24 h. As such, these sponges withheld higher quantities of SNAP after 4 h, diffusing 32, 47, and 59% of their total SNAP (Table S2).

This discrepancy can be attributed to the variations in porosity among the sponges, with the higher porosity of the 83 and 88% porous sponges facilitating faster liquid penetration and more efficient SNAP extraction from the polymer matrix. Conversely, the less porous sponges retained higher quantities of SNAP for longer periods. The observed change in color during the study provided a visual indicator of SNAP extraction. Sponges that diffused greater than 90% of their SNAP content (77, 83, and 88% porous sponges) reverted to their original white color, with little to no visible green pigmentation remaining.

#### 3.2.3 Measuring Real-Time NO Release under Physiological Conditions

Nitric oxide release from SNAP sponges was assessed under physiologically relevant conditions for 4 h based on the significant diffusion of SNAP after 4 h from the 83 and 88% porous sponges. Given that many biomedical devices come into contact with bodily fluids, including blood, sponge samples were exposed to 37 °C PBS to evaluate their NO release kinetics under physiological conditions. This assessment is crucial for determining the sponges’ potential utility in medical applications. While exposed to these conditions, the SNAP sponges demonstrated a steady increase in NO release (Figure 3A). The cumulative NO release from samples was calculated over the entire 4 h period (Figure 3B). Among the sponge types, the 88% porous sponge exhibited the highest rates of NO release throughout the study duration, accumulating a total of 50.72 nmol of NO per mg of sponge, respectively. The trends observed in NO release within the different sponge types were strongly correlated with the SNAP concentration present in the sponges, highlighting the role of porosity and SNAP loading in governing the release kinetics. The continuous release of NO from the sponges correlates with the diffusion of SNAP in solution signifying that NO is being quickly depleted from the sponges.

**Figure 3:**
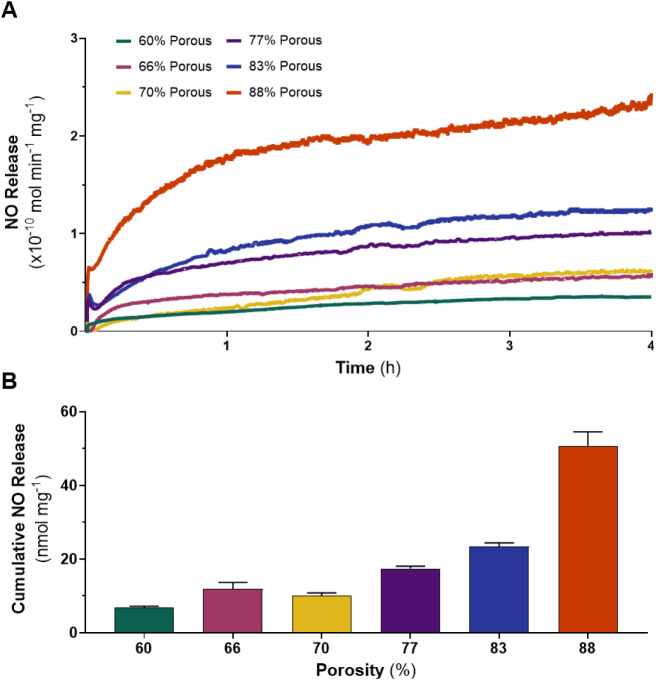
NO release activity of SNAP-incorporated PDMS sponges. (A) Representative NO release profiles of SNAP-incorporated PDMS sponges in physiological conditions over 4 h. (B) Cumulative production of NO from SNAP-incorporated sponges in physiological conditions after 4 h exhibited increased release rates as porosity increases. Data are presented as mean ± SEM (*n* = *3*).

The presence of void space within the sponge directly impacts the ability of SNAP to interact with liquids and facilitate NO release. In practical scenarios like a wound dressing application, the 60–70% porous sponges would encounter challenges in effectively absorbing exudate from the surrounding environment. These challenges stem from both PDMS’s hydrophobic nature and their comparatively lower porosity, making it a challenge to achieve liquid absorption without oversaturation. In contrast, the 83 and 88% porous sponges may not be ideal for applications that require sustained NO release for extended antimicrobial activity.

By the end of the study duration, the 83 and 88% sponges lost much of their green pigmentation, correlating with the amount of SNAP diffused. The porous structure facilitates a more extensive diffusion pathway for NO, enabling higher release rates. These findings highlight the importance of optimizing the sponge’s porosity to achieve desired release characteristics for specific applications. Overall, the study provides valuable insights into the NO release behavior of SNAP-incorporated PDMS sponges.

To further investigate NO release from the SNAP-incorporated sponges, EDS was performed before and after exposure to physiological conditions for 4 h following supporting methods (Section S1.4). Elemental maps of sulfur (S) and nitrogen (N) were used to evaluate the relative loss of nitrogen, which is cleaved from the S–NO bond during NO release, while sulfur remains within the residual structure. As shown in Figure S6, all sponge types exhibited an increased S:N atomic ratio after swelling, supporting that NO release occurred during this incubation period. Notably, the increase in S:N ratio was more pronounced in sponges with higher porosity, suggesting that increased porosity enhances NO release, as a result of improved fluid diffusion and greater SNAP accessibility within the polymer matrix.

Although SNAP contains a 1:1 stoichiometric ratio of S to N, the initial EDS measurements of dry, unswollen sponges showed slight deviations from this ratio. This is likely due to a combination of factors, including the surface-sensitive nature of EDS, heterogeneous SNAP distribution within the porous network, and differences in elemental detectability, particularly for nitrogen. Nevertheless, the relative change in the S:N ratio before and after swelling provides a reliable qualitative indicator of NO release behavior, consistent with observed porosity-dependent trends.

### 3.3 Bacterial Viability

The antibacterial efficacy of NO-releasing sponges was assessed in a 4 h bacterial study, using E. coli and S. aureus as representative Gram-negative and Gram-positive bacteria. These bacterial strains were selected for their relevance in biomedical contexts; E. coli is commonly linked to urinary tract and surgical site infections, while S. aureus is a leading cause of wound and implant-related infections.^32^ The choice of microbes was also based on their existing antibiotic-resistant strains, highlighting the potential for the emergence of further resistant variants.^33^ Consequently, there is a pressing need for alternative treatment methods that can effectively eliminate microbes without the risk of resistance development. The application of NO ensures that antibiotics can be reserved for the most severe cases. Therefore, this study aimed to evaluate the sponges’ effectiveness in preventing bacterial colonization on the surface and inhibiting further attachment of planktonic bacteria. This targeted approach provides potential applicability of the NO-releasing sponges in biomedical settings, where the prevention and treatment of infections caused by Gram-negative and Gram-positive bacteria are of paramount importance.

During the bacterial adherence study, bacteria were exposed to control and SNAP-incorporated sponges for 4 h, during which high rates of NO release were observed from the 88% porous sponge (Figure 3B). The results revealed an increase in bactericidal activity against both E. coli (Figure 4A) and S. aureus (Figure 4C) with higher sponge porosity. Bacterial viability reduction was assessed by calculating the CFUs mg^–1^ and comparing SNAP-incorporated sponges with their corresponding controls without SNAP (*p* < 0.05 for all sponge types) (Figure S7). The 60% porous sponge exhibited a 1.48-log reduction in viable E. coli, while the 88% porous sponge demonstrated a 2.45-log reduction. Similarly, the 60% porous sponge displayed a 1.07-log reduction in S. aureus viability, whereas the 88% porous sponge exhibited a 2.64-log reduction in bacterial viability, respectively.

**Figure 4:**
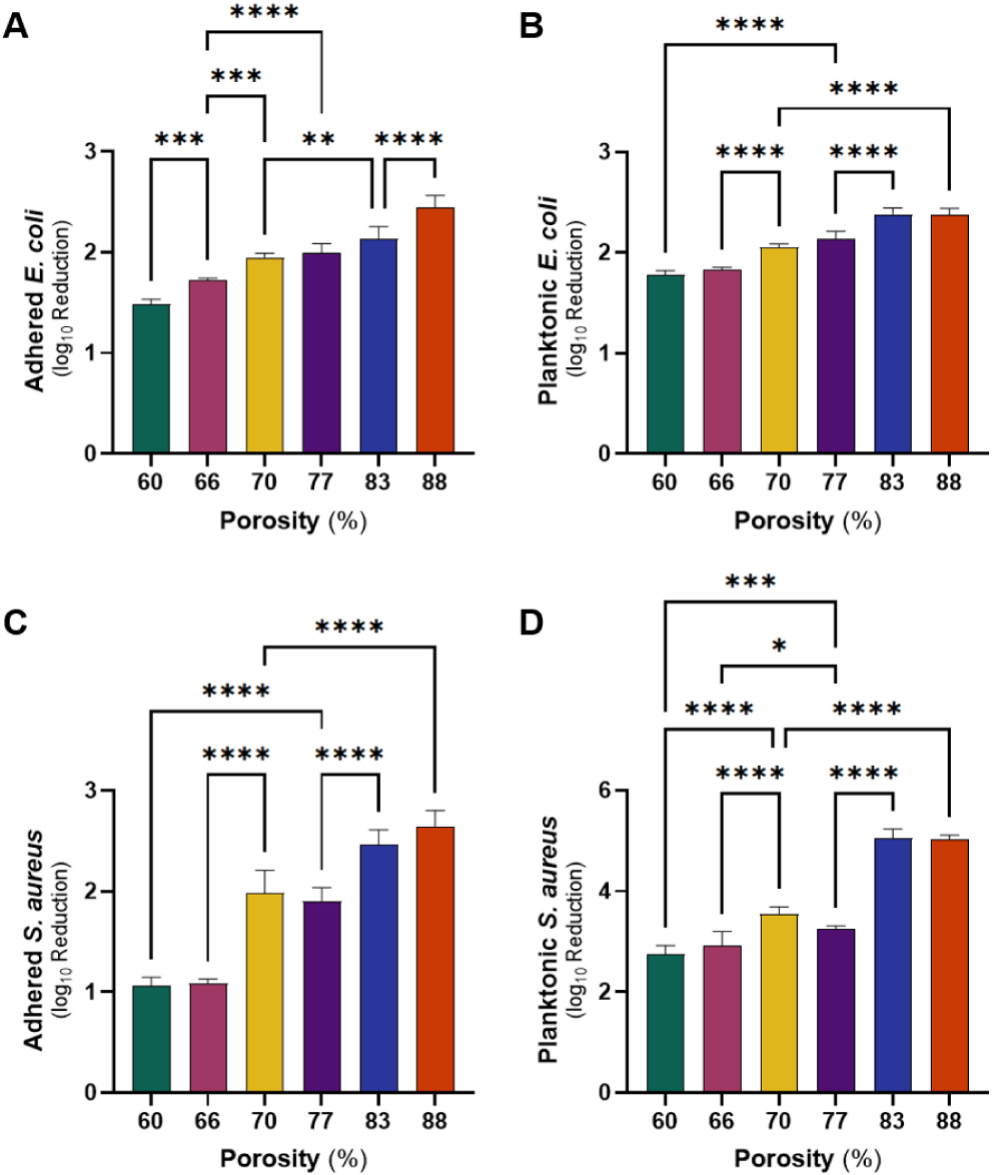
Bacteria reduction of SNAP-incorporated PDMS sponges with respect to control sponges against adhered (A) and planktonic (B) E. coli and adhered (C) and planktonic (D) S. aureus after 4 h exposure to bacteria. Data are presented as mean of two biological replicates ± SEM (*n* = *6*).

Simultaneously, viable planktonic bacteria were investigated after being exposed to control and SNAP-incorporated sponges for 4 h, during which high SNAP diffusibility was observed from the 83 and 88% porous sponges (Figure 2C). Correspondingly, an increase in bactericidal activity against both E. coli (Figure 4B) and S. aureus (Figure 4D) with higher sponge porosity was observed. Bacterial viability reduction was assessed by calculating the CFUs/mL normalized to the sponge weight (mg^–1^) and comparing SNAP-incorporated sponges with their corresponding controls without SNAP (*p* < 0.05 for all sponge types) (Figure S7). A 1.78- and 2.74-log reduction in E. coli and S. aureus viability was observed after exposure to the 60% porous sponge, while the 88% porous sponge exhibited 2.38- and 5.02-log reductions in viable E. coli and S. aureus viability, respectively.

The observed bactericidal activity against both E. coli and S. aureus yielded intriguing insights into the role of porosity in bacterial eradication. Despite noticing variations in SNAP loading, leaching, and NO release among the different sponge types, the bactericidal activity did not reveal statistically significant differences across all sponges (Tables S3–S6). This suggests that the bactericidal effect of the SNAP-incorporated sponges may be primarily driven by the concentration of NO rather than the porosity of the sponges. For clarification, NO concentrations between 6.88 and 11.82 nmol mg^–1^ correlate with 1.6, 1.8, 1.07, and 2.83 log reductions for adhered and planktonic E. coli and S. aureus, respectively. Similarly, NO concentrations between 10.12 and 17.41 nmol mg^–1^ correlate with 1.97, 2.09, 1.94, and 3.39 log reductions, and concentrations between 23.35 and 50.72 nmol mg^–1^ correlate with 2.29, 2.38, 2.55, and 5.03 log reductions for the same bacterial species. These findings align with prior research, indicating that the concentration of NO achieved in porous materials plays a pivotal role in determining their antimicrobial efficacy against various bacterial strains.^34^

Notably, a discernible trend persisted in both adhered and planktonic experiments as sponge porosity increased. Specifically, the 83 and 88% porous sponges consistently demonstrated significant bactericidal activity against E. coli when compared to the other sponge types with the 88% porous sponge preventing more E. coli adhesion than the 83% porous sponge. Similarly, the 70 and 77% porous sponges exhibited analogous bacterial killing efficiency, showing significance over the 60 and 66% porous sponges.

In the case of S. aureus, the 83 and 88% porous sponges exhibited significantly higher bactericidal activity compared to the other sponge types. Following a similar trend to that of E. coli, the 70 and 77% porous sponges demonstrated significant killing when compared to the 60 and 66% porous sponges. Although similar trends are seen within both bacterial strains and experiments, an overall higher killing efficiency is seen against S. aureus than E. coli, with an emphasis on the planktonic bactericidal activity. This divergence in antibacterial performance between bacterial strains can be explained by the distinct cell wall and membrane compositions of Gram-negative E. coli and Gram-positive S. aureus bacteria.^35^ These disparities likely result in varying requirements for effective bactericidal activity, with certain sponge porosities proving more effective against one bacterial strain over the other. Importantly, this explanation also sheds light on the overall increase in bactericidal activity exhibited by all sponges against planktonic S. aureus when compared to E. coli.

As porous scaffolds have been shown to facilitate cell adhesion and proliferation due to more attachment sites,^36^ the porous nature of the PDMS sponge would be expected to act similarly. As the porosity of the sponge increased, more bacterial growth was observed on control samples in both adhered and planktonic studies (Figures S8–S11, Tables S7–S10). Bacterial adhesion to the sponge surface aided in the increased concentration of CFU in adhesion and in the free-floating bacteria. The variations observed between the two bacteria can be correlated to the bacteria having different mechanisms of adhesion and proliferation.^37^ Nonetheless, this increase in both E. coli and S. aureus adhesion to control sponges highlights the significant bactericidal effect of incorporating SNAP into the sponge. Although more bacteria adhered to the control sponges, increasing the SNAP loading significantly inhibited adhesion as porosity increased. These findings reveal the complex interplay between NO release, bacterial strains, and sponge porosity, emphasizing the importance of understanding the specific mechanisms of action and concentration requirements for effective bactericidal activity.

### 3.4 Cytocompatibility of SNAP Sponges

The cytocompatibility of SNAP-incorporated PDMS sponges (60, 77, and 89%) was evaluated via indirect exposure of L929 mouse fibroblasts to sponge leachates via supporting methods (Section S1.5). Cell viability was assessed after 24 h of exposure to 100, 50, and 10% diluted leachates using the MTT assay.

All sponge types at 100 and 50% leachate concentrations resulted in cytotoxic activity, with cell viabilities falling below the 70% threshold defined by ISO 10993-5 for noncytotoxicity (Figure S12). Notably, only the 10% diluted leachates of the 60% porous sponges maintained cell viability above 70%, indicating cytocompatibility under diluted conditions. In contrast, the 10% leachates from the 77 and 89% porous sponges resulted in cytotoxic responses, likely due to their higher SNAP content and faster leaching behavior.

## 4 Discussion

Fine-tuning sponge porosity can be achieved by modifying the porogen concentration during assembly. Sponges were fabricated using various amounts of NaCl to create 7 sponges, each possessing a distinct level of porosity. The observed porosity trend is consistent with previous literature in which an increase in porogen concentration increases the porosity of the material,^1,38^ with obtained values closely aligning with those reported for PDMS sponges fabricated with NaCl.^2^ These findings indicate that the porogen concentration has control over the sponge’s porosity, which is particularly important for achieving desired properties. As all sponge types underwent an identical fabrication process, no significant changes in the macroscopic network structure were observed across the formulations. This observation implies that the change in porosity can be attributed to an increase in the quantity of pores rather than alterations in pore sizes. Furthermore, the average pore size of each sponge type closely aligns with previously reported studies that fabricated PDMS sponges using NaCl as the porogen.^2,39^

The mechanical integrity of the sponges is an essential characteristic in selecting the appropriate porosity for specific antibacterial applications. For applications such as bone tissue engineering or orthopedic implants, stronger materials are necessary to withstand appropriate loads. Conversely, for disinfection and wound dressing applications, particularly those involving soft tissue, more flexible sponges are preferred to better conform to the surrounding environment. The effect of porosity and SNAP incorporation on compressive strength was evaluated, showing that increasing porosity reduced compressive strength, while SNAP incorporation increased the compressive modulus across all sponge types tested. Although this phenomenon has not been previously observed in sponge materials, SNAP has not been reported to significantly impact mechanical properties in other materials. However, in porous hydrogels, SNAP incorporation led to a slight increase in compressive modulus.^40^ This discrepancy may be attributed to SNAP crystallizing within the pores, which could contribute more significantly to the compressive modulus in porous sponges.^41^ Furthermore, as porosity increased, the percent increase in compressive modulus after SNAP incorporation became more pronounced, with the 60, 77, and 89% porous sponges exhibiting increases of 33.1%, 161%, and 356%, respectively. These findings further suggest that higher porosity facilitates greater SNAP incorporation and embedding within the pores, resulting in enhanced mechanical reinforcement.

While PDMS is inherently hydrophobic and not traditionally used in wound dressings, the incorporation of SNAP and the introduction of tunable porosity significantly enhanced the swelling behavior of the sponges in biologically relevant media. In particular, the 77 and 89% porous sponges reached absorption levels up to 400–500%, values that are comparable with other hydrophobic wound dressings, which typically absorb 300–1000% of their dry weight.^42,43^ On the other hand, hydrophilic wound dressings, such as hydrogels and superabsorbent polymers, can achieve much higher swelling capacities, absorbing up to 10–60 times their dry weight, often within just a few hours.^44–47^ While the PDMS-based sponges demonstrated notable absorption (up to ∼5 times their weight), their slower and more moderate swelling profile may limit their applicability for high-exudate, deep, or chronic wounds, where rapid fluid uptake is critical. However, their ability to swell in biologically relevant fluids and incorporate active agents may still render them suitable for smaller, lower-exudate wounds or applications where controlled fluid handling is desired. Among the sponge formulations, the 60% porous SNAP sponge may be particularly promising. Despite its lower porosity, it demonstrated a meaningful increase in fluid absorption, especially in simulated wound fluid, and showed more moderate and controlled swelling behavior compared to higher-porosity variants. This restrained swelling could help preserve material integrity and ensure localized therapeutic delivery without excessive expansion or premature drug loss. Additionally, the lower porosity may contribute to slower fluid ingress, potentially supporting more sustained NO release in wound environments.

Sponge materials are widely used in applications such as wound healing and water treatment, and they have been engineered with antimicrobial properties to enhance their effectiveness. Although these materials exhibit strong antimicrobial activity,^48^ the influence of porosity on their pharmacokinetic and antimicrobial properties has not been thoroughly explored. This study establishes direct correlations between the porosity of PDMS sponges and the antimicrobial effectiveness of NO-releasing sponges loaded with SNAP. Herein, a SNAP concentration of 25 mg/mL was selected based on prior evidence in silicone tubing, demonstrating its ability to yield physiologically relevant NO release in both *in vitro* and *in vivo* settings.^23,49^ At higher concentrations of SNAP (50 and 125 mg/mL), the sponges became brittle due to excessive SNAP crystallization around the material.

The porous nature of the PDMS sponge material significantly enhanced SNAP loading efficiency compared to previous studies using various silicone-based materials. These prior works achieved a mere ∼5 wt % loading with a 125 mg/mL SNAP solution in THF.^31,50^ However, the 88% porous sponge exhibited a significant increase in SNAP loading (23 wt %), surpassing previous reports of SNAP loading in various polymers.^19,51,52^

Diffusion of antimicrobial agents into a solution provides a practical scenario for applications such as wound healing, where the material comes into contact with blood. The diffusion of SNAP in PBS offers initial insights into the release behavior of PDMS sponges with varying porosity. Nonporous SNAP-swelled PDMS films have been reported to diffuse less than 1% of its SNAP over 24 h, reflecting the slower penetration of PBS into the material and reduced hydrolytic cleavage of NO.^53^ However, similar patterns have been observed in drug-release behavior in porous materials.^54,55^ These findings open a broad spectrum of potential applications in humid conditions, ranging from the need for sustained, low quantity NO release over an extended period to instances requiring rapid, high-volume NO bursts. Extended NO release can be particularly beneficial for certain biomedical devices intended for long-term clinical use, such as tunneled dialysis catheters, offering prolonged hemocompatibility and biocompatibility to prevent thrombosis and infection.^56^ Additionally, SNAP-incorporated materials have been coated with one or multiple thin polymer layers to reduce SNAP diffusion.^24^ These approaches offer potential strategies to improve the retention of SNAP within sponge materials, particularly addressing the challenge posed by less porous sponges that contain limited quantities of the donor molecule. This presents advantages for applications mentioned earlier, specifically those requiring continuous NO release, such as preventing thrombosis in long-term catheters and similar medical devices.

The observed rapid leaching of SNAP from highly porous sponges suggests that a portion of the SNAP may be loosely associated with the PDMS matrix rather than fully embedded. While this results in a rapid initial release, it also enhances antimicrobial efficacy, as demonstrated in both adhered and planktonic bacterial assays.

Previously reported NO release from different sponge materials, such as collagen, have shown short release durations.^17^ These collagen sponges, incorporated with *S*-nitrosoglutathione, released approximately 0.64 nmol mg^–1^ of NO in almost 2 h, releasing levels of NO comparable to that of the endothelial to promote wound healing.^57^ The SNAP-incorporated sponges released significantly higher amounts of NO, with even the 60% porous sponge releasing approximately 6.88 nmol mg^–1^ of NO. The observed NO release over 4 h showcases the potential of PDMS sponges for applications requiring high quantities of NO release in a short period of time, such as disinfection of medical devices and infection prevention in open wounds. Recently, PDMS sponges have been modified and used in applications such as medical devices, conductive materials, and horticulture.^2,58,59^ These applications can be further improved by incorporating NO donors like SNAP, which enhance the material’s antithrombotic and antibacterial properties. The trend in NO release performance, ranging from 60 to the 88% porous sponge, suggests that porosity plays a role in influencing release kinetics through increasing SNAP-loading and altering diffusion pathways. To increase the NO release levels and potentially prolong release rates, SNAP could be covalently bound to the PDMS polymer, creating a tighter bond to the material and reducing the diffusion of SNAP over time.^22^

Nonetheless, the significant antibacterial activity exhibited by the NO-releasing sponges highlights their potential for applications in various biomedical settings, where preventing and treating infections caused by E. coli and S. aureus are critical. The 83 and 88% porous sponges displayed superior antibacterial activity compared to other NO-releasing materials. In comparison, NO-releasing PDMS films, using a similar SNAP swelling process (25 mg/mL), exhibited log reductions of 0.77 and 1.42 against adhered E. coli and S. aureus after 24 h of exposure, respectively.^60^ Moreover, silicone catheters swelled with SNAP (50 mg/mL) exhibited 1.96- and 1.70-log reductions in viable adhered E. coli and S. aureus, respectively, and 0.74- and 1.11-log reductions against planktonic E. coli and S. aureus following a 24 h exposure.^61^ It is worth noting that the 70–88% porous SNAP-incorporated sponges achieved log reductions equivalent to or exceeding those of previously reported SNAP-incorporated materials against both E. coli and S. aureus. These studies provide valuable insights into the potential of NO-releasing PDMS sponges as highly effective antibacterial materials against common bacterial pathogens. The findings highlight the significance of fine-tuning NO concentration and sponge porosity to obtain the desired bactericidal efficacy tailored for specific applications. Further research endeavors can center on enhancing the mechanisms of NO action, tailoring them to specific uses such as wound healing, infection prevention, and medical device disinfection. By understanding and refining the intricate interplay between NO release, bacterial strains, and sponge porosity, these sponges can be further developed for targeted and highly effective antimicrobial applications in diverse biomedical contexts.

The cytocompatibility results highlight the critical role of porosity in determining the biological safety of SNAP-incorporated PDMS sponges, particularly due to its influence on rapid leaching and subsequent cellular responses. While all sponge formulations were capable of releasing NO, the combination of rapid NO release and increased SNAP leaching, particularly at higher porosities, likely contributed to elevated concentrations of cytotoxic species and byproducts in the surrounding environment. This effect was most pronounced in the 77 and 89% porous sponges, where even highly diluted leachates significantly reduced fibroblast viability. Among the tested conditions, the 10% leachate from the 60% porous sponge met the cytocompatibility threshold, suggesting that lower porosity may provide better control over leaching and mitigate cytotoxic effects. However, further optimization is needed before these materials can be considered suitable for wound healing applications. To improve cytocompatibility, future strategies may include reducing SNAP loading, engineering surface coatings or diffusion barriers to modulate release kinetics, or incorporating additives that stabilize NO release while minimizing the generation of harmful leachates.

Recently, NO-releasing sponge materials have been successfully fabricated and investigated to enhance their antimicrobial properties in wound healing applications.^62–64^ These sponges, however, measure nitrite via a Griess Assay, which indirectly quantifies NO but lacks real-time measurement and can be influenced by other nitrite sources, making it less accurate than direct NO measurement by a NOA.^65^ Agents such as antibiotics and essential oils have also been incorporated into sponges to provide the material with antimicrobial release mechanisms.^66,67^ Combining NO releasing technology with other antimicrobial agents in a sponge material may provide enhanced antimicrobial efficacy against various microbes, such as fungi, in addition to the bacteria tested herein, enhancing the applicability of these materials.

## 5 Conclusions

This study employed a simple and sustainable template extraction technique using hot water and NaCl as a porogen to fabricate PDMS sponges with porosities ranging from 60 to 89% and NO-releasing properties. The ability to modify the porosity of the sponges was demonstrated through the loading and diffusion of SNAP, with increasing porosity resulting in higher SNAP loading due to a larger void space-to-PDMS polymer ratio. The release behavior of NO under physiological conditions and the diffusibility of SNAP aligned with the results of SNAP loading, further confirming the relationship between porosity and NO release. The 60% porous sponge released approximately 6.88 nmol mg^–1^ of NO within 4 h, correlating with a 1.48- and 1.78-log reduction in viable adhered E. coli and S. aureus and a 1.06- and 2.74-log reduction in viable planktonic E. coli and S. aureus. Similarly, the 88% porous sponge released approximately 50.72 nmol mg^–1^ of NO within 4 h, correlating with a 2.45- and 2.64-log reduction in viable adhered E. coli and S. aureus and a 2.38- and 5.02-log reduction in viable planktonic E. coli and S. aureus. Although further optimization is needed for cytocompatibility, the sponges ability to restrict both E. coli and S. aureus attachment and growth demonstrates their potential for antibacterial applications, particularly in wound healing scenarios and the disinfection of biomedical devices.

Furthermore, the findings of this study offer an innovative approach to address the rising global health challenge of antibiotic resistance in bacteria. The ability to fine-tune the porosity of the sponge and achieve tailored antibacterial properties can potentially reduce the reliance on conventional antibiotics, mitigating the risk of bacterial resistance development. This work provides valuable insights into the relationship between porosity, NO release, and antibacterial activity in PDMS sponges. Customization of material properties opens avenues for developing advanced biomedical devices, wound healing strategies, and infection control measures. These broader impacts extend to the realm of antibacterial resistance and healthcare-associated infections, where NO-releasing sponges offer a sustainable and adaptable solution to improve patient outcomes and enhance the field of biomedical engineering.

## Supplementary Material



## Notes

SNAP characterization by NMR; static water contact angle measurements; sulfur elemental mapping via EDS; mechanical properties via compressive testing; swelling behavior in simulated wound fluid; statistical significance values for SNAP loading; quantitative SNAP leaching data; S:N elemental ratio analysis by EDS; statistical analysis of antibacterial efficacy; cytocompatibility data with MTT assay results (PDF)
