# Supplementary material for: Nitric Oxide-Releasing Polydimethylsiloxane Sponges with Tunable Porosity
Source: ACS Appl Mater Interfaces. 2025 Jun 9;17(24):35262–74. doi: 10.1021/acsami.5c06963 (PMC12186219; doi:10.1021/acsami.5c06963)
Supplement: Supplementary file 1 [file am5c06963_si_001.pdf]

Supporting Information

Nitric Oxide-Releasing Polydimethylsiloxane Sponges with Tunable Porosity

Adam Brooks Goodman[1], Manjyot Kaur Chug[1], Natalie Crutchfield[1], Hitesh Handa[1,2], Elizabeth J. Brisbois*[1]

[1] School of Chemical, Materials, and Biomedical Engineering, College of Engineering, University of Georgia, Athens, Georgia, 30602, USA

[2] Department of Pharmaceutical and Biomedical Sciences, College of Pharmacy, University of Georgia, Athens, Georgia, 30602, USA

Corresponding Author:

Prof. Elizabeth J. Brisbois
School of Chemical, Materials, and Biomedical Engineering
College of Engineering
University of Georgia
302 East Campus Rd
Athens, GA 30602
Telephone: 706-542-1243
E-mail: ejbrisbois@uga.edu

Authors

Adam Brooks Goodman - School of Chemical, Materials, and Biomedical Engineering, College of Engineering, University of Georgia, Athens, Georgia, 30602, United States

Manjyot Kaur Chug - School of Chemical, Materials, and Biomedical Engineering, College of Engineering, University of Georgia, Athens, Georgia, 30602, United States

Natalie Crutchfield - School of Chemical, Materials, and Biomedical Engineering, College of Engineering, University of Georgia, Athens, Georgia, 30602, United States

Hitesh Handa - School of Chemical, Materials, and Biomedical Engineering, College of Engineering, University of Georgia, Athens, Georgia, 30602, United States;

Department of Pharmaceutical and Biomedical Sciences, College of Pharmacy, University of Georgia, Athens, Georgia, 30602

1. Supporting Methods

S1.1 Wettability Measurements

The effect of SNAP-incorporation on the surface wettability of the sponges was assessed using an Ossila Contact Angle Goniometer (Ossila, Sheffield). A 10 µL droplet of water was placed onto the surface of control and SNAP sponges (60, 77, 89% porous) and the static contact angle was obtained by calculating the average of at least four samples (approximately 6 mm in diameter and 7 mm in height).

S1.2 Compressive Modulus

Uniaxial compression testing was performed using a Discovery HR-2 DMA (TA Instruments, New Castle, DE). Control and SNAP sponges (60, 77, 89% porous) were compressed from 10% to 40% strain at a rate of 50 µm s^{-1}. The mechanical testing was completed with cylindrical samples (approximately 6 mm in diameter and 7 mm in height). The diameter and thickness of each sample were measured from at least three different starting points along its length with an accuracy of 0.025 mm. The minimum value of the cross-sectional area, along with the length of each sample, was recorded for further analysis. The stress-strain relationship was analyzed to determine the modulus of elasticity.

S1.3 Fluid Absorption Using Simulated Wound Medium

The swelling capacity of control and SNAP-incorporated sponges (60%, 77%, and 89%) was evaluated over 48 h to investigate their potential for wound healing applications. Sponge samples (approximately 6 mm in diameter and 5 mm in height) were immersed in 1 mL of either phosphate-buffered saline (PBS) or simulated wound fluid (SWF), composed of a 1:1 mixture of PBS and fetal bovine serum (FBS). To facilitate liquid uptake in the hydrophobic sponges, samples were placed on a rocker at room temperature throughout the study. At predetermined time points, sponges were carefully removed, blotted to remove excess surface fluid, and weighed. The swelling (absorption) capacity was then calculated using Equation S1.

$$Absorption\ Capacity\ (\%) = (\frac{m_s - m_o}{m_o}) * 100\% \quad \text{(S1)}$$

Where m_s is the swollen mass of the sample over time and m_o is the dry mass of the sponge.

S1.4 Elemental Analysis of NO Release via EDS

To further evaluate NO release from SNAP-incorporated PDMS sponges, elemental analysis was performed using energy-dispersive X-ray spectroscopy (EDS) to quantify relative changes in sulfur (S) and nitrogen (N) content. Sponges with 60, 77, and 89% porosity were first analyzed in their dry state (pre-swelling) to establish baseline elemental composition. Subsequently, the sponges were submerged in 1 mL PBS containing 100 µM EDTA at 37 °C for 4 h to promote NO release. After incubation, the samples were dried under vacuum and analyzed using the same EDS conditions as the dry controls. Prior to imaging, the samples were sputter-coated with a 10 nm layer of gold–palladium using a Leica sputter coater (Leica Microsystems). EDS was conducted on a field emission scanning electron microscope (FEI Teneo, FEI Co.) to detect the presence and distribution of S and N in the SNAP-incorporated sponges. The S:N atomic ratio was calculated before and after swelling using ImageJ software (National Institutes of Health, Bethesda, MD) to assess the relative depletion of nitrogen due to cleavage of the S-NO bond and subsequent NO release.

S1.5 Evaluation of Cytocompatibility

Cells were revived from cryopreserved stocks using complete media for each cell line following the manufacturers' recommendations. Mouse fibroblast L929 cells were cultured in fibroblast basal medium supplemented with fetal bovine serum (10%) and penicillin–streptomycin (1%). Cells were incubated at 37 °C under a 5% CO_2-humidified atmosphere, treated with clean media every 48 h, and grown to no greater than 80% subconfluency. For the controlled release leachate testing, cells were detached via enzymatic treatment with trypsin (0.05% supplemented

with 5 mM EDTA), centrifuged to collect the cell pellet (500 RCF, 5 min), and resuspended to achieve a seeding density of 10,000 cells/well in culture-treated 96-well plates.

The cytocompatibility of SNAP-incorporated PDMS sponges was evaluated using an indirect extract method in accordance with ISO 10993-5:2009 guidelines. Sponges (60, 77, or 89% porous) were sterilized under UV light for 15 min per side, then incubated in complete Dulbecco's Modified Eagle Medium (DMEM, VWR, Radnor, PA, USA) supplemented with 10% FBS and 1% penicillin-streptomycin for 4 h at 37 °C to generate leachates. Mouse fibroblast L929 cells were seeded in 96-well plates at a density of 10,000 cells/well and allowed to attach for 24 h. After incubation, culture media were replaced with 100 µL of collected leachates diluted to 100, 50, and 10% concentrations using fresh media. Cells were exposed to leachates for 24 h, followed by viability analysis using the MTT assay. A working solution of MTT (3-(4,5-dimethylthiazol-2-yl)-2,5-diphenyltetrazolium bromide; Sigma-Aldrich) at 0.5 mg/mL was added to each well and incubated for 4 h at 37 °C under 5% CO_2. The resulting formazan crystals were solubilized using dimethyl sulfoxide (DMSO), and absorbance was measured at 570 nm with a reference reading at 650 using a microplate reader (BioTek Synergy HTX, Agilent). Cell viability was expressed as a percentage relative to untreated control cells (n = 4 technical repeats across one independent passage).

2. Supporting Results

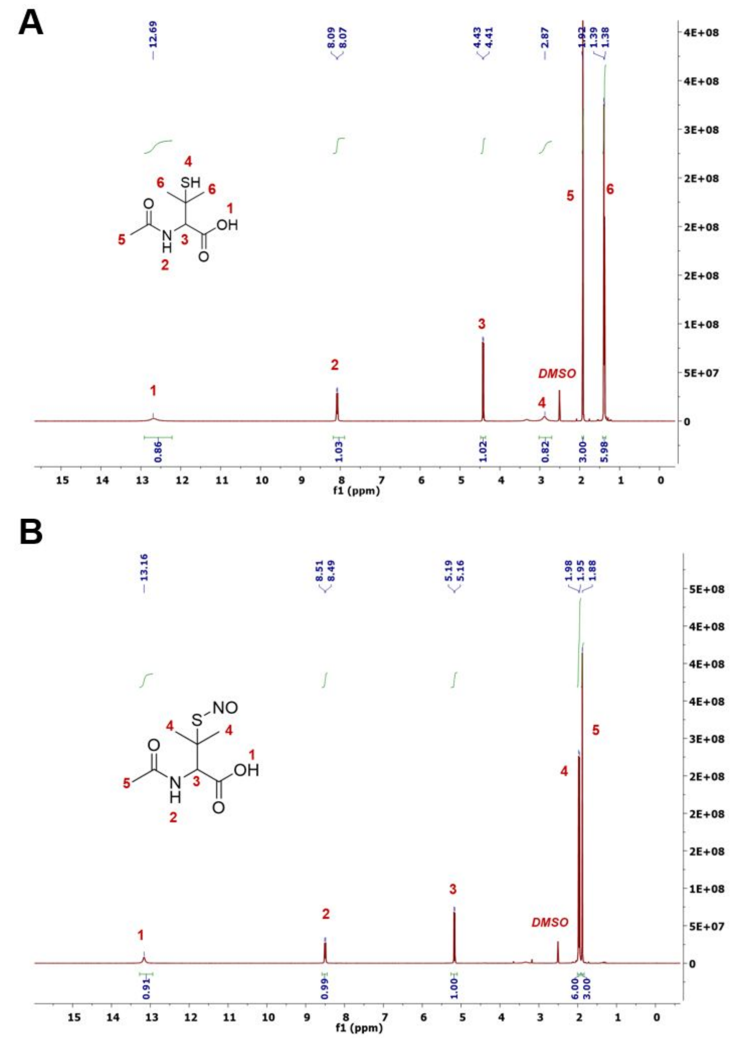

Figure S1. ^{1}H NMR spectra of **(A)** 2-acetamido-3-methyl-3-sulfanylbutanoic acid (NAP) and **(B)** 2-acetamido-3-methyl-3-(nitrosothio)butanoic acid (SNAP) in DMSO-d_6.

2-acetamido-3-methyl-3-sulfanylbutanoic acid (NAP):

^{1}H NMR (400 MHz, DMSO-d_6) δ 12.69 (s, 1H), 8.08 (d, J = 9.1 Hz, 1H), 4.42 (d, J = 9.1 Hz, 1H), 2.87 (s, 1H), 1.92 (s, 3H), 1.39 (d, J = 5.8 Hz, 6H).

The ^{1}H NMR spectrum of NAP was recorded in DMSO-d_6 at 400 MHz. A singlet was observed at δ 12.69 ppm, corresponding to the acidic proton from the carboxylic acid group. A doublet at δ 8.08 ppm and δ 4.42 ppm represent the amide NH and nearby methine protons. A singlet at δ

2.87 ppm depicts the -SH (thiol) proton. The methyl group attached to the acetamido side chain produced a singlet at δ 1.92 ppm. A doublet at δ 1.39 ppm, integrating for six protons, was observed for the two methyl groups on the tertiary carbon.

2-acetamido-3-methyl-3-(nitrosothio)butanoic acid (SNAP):

^{1}H NMR (400 MHz, DMSO-d_6) δ 13.16 (s, 1H), 8.50 (d, J = 9.5 Hz, 1H), 5.17 (d, J = 9.5 Hz, 1H), 1.97 (d, J = 10.0 Hz, 6H), 1.88 (s, 3H).

The ^{1}H NMR spectrum of SNAP confirms the successful conversion of the NAP thiol group (-SH) to a nitrosothiol (-SNO). In the spectrum, the peak for the thiol proton at δ 2.87 ppm seen in NAP has disappeared. A new set of peaks is observed, with slight shifts compared to the original compound. The amide (NH) and its neighboring methine proton now appear at δ 8.50 ppm and δ 5.17 ppm due to the introduction of the electron-withdrawing -SNO group, changing the electronic environment for nearby protons. The carboxylic acid proton also appears slightly shifted at δ 13.16 ppm. The methyl protons show at δ 1.97 ppm (doublet, 6H) and δ 1.88 ppm (singlet, 3H), consistent with the expected structure. These changes confirm the successful formation of SNAP.

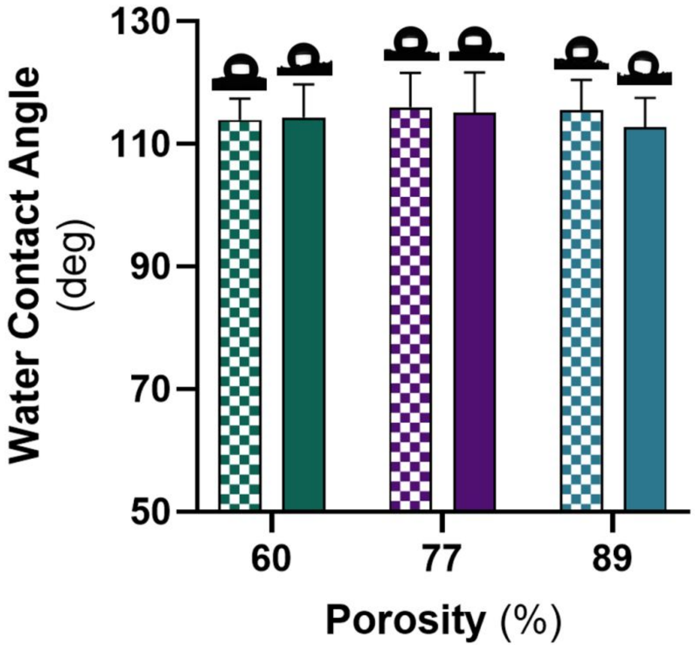

Figure S2. Static water contact angle measurements of control and SNAP sponges indicating no significant change in wettability with increasing porosity or SNAP incorporation. Data are presented as mean ± STDV ($n \geq 4$).

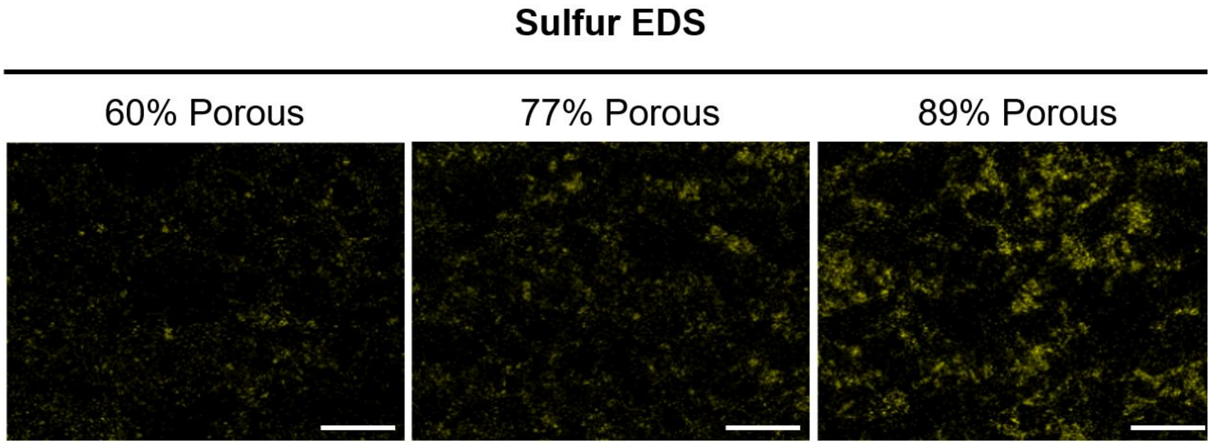

Figure S3. Energy-dispersive X-ray spectroscopy (EDS) analysis of 60, 77, and 89% porous sponges after SNAP incorporation. Sulfur (S) in the S-NO bond identifies the presence of SNAP in cross-sectional images. Scale bars represent 1mm.

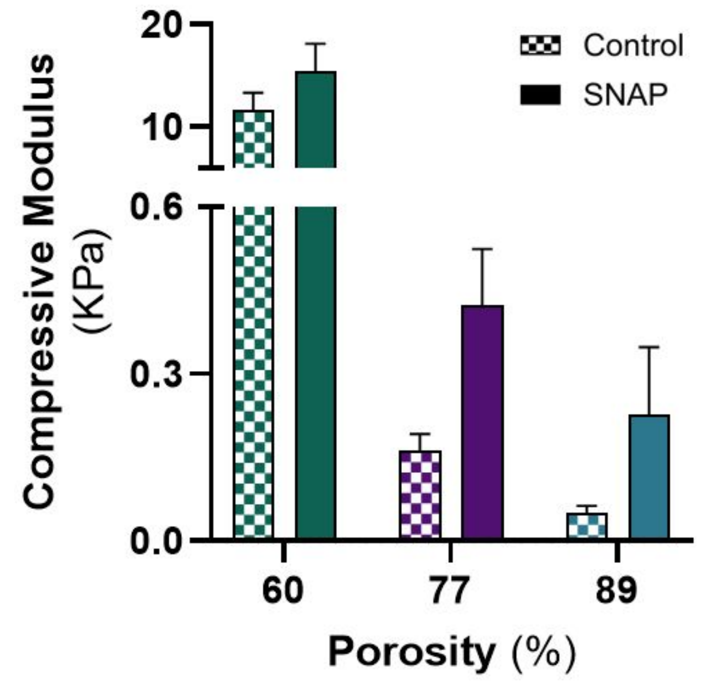

Figure S4. Compressive modulus of control and SNAP sponges indicating that void space present within the material effects the mechanical integrity of the sponge. Data are presented as mean ± STDV ($n \geq 5$).

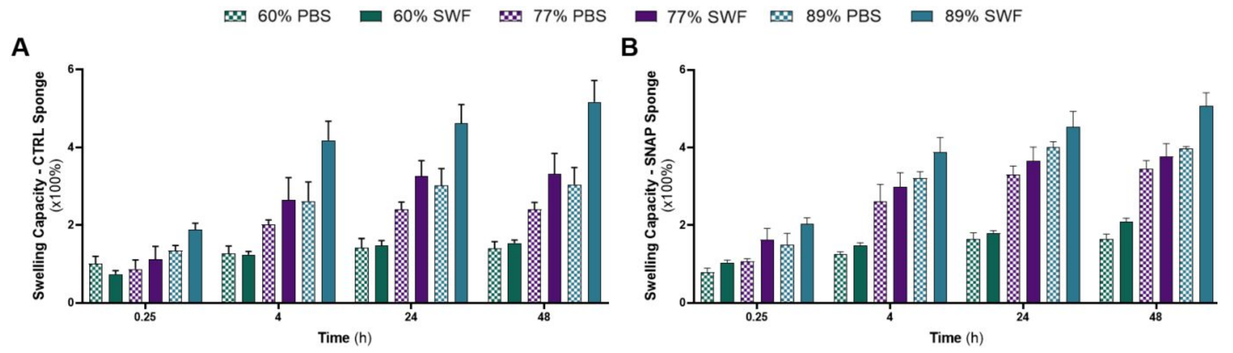

Figure S5. Swelling behavior of control **(A)** and SNAP-incorporated **(B)** sponges (60%, 77%, and 89%) over 48 h in PBS and SWF (1:1 PBS:FBS). Swelling capacity was determined gravimetrically using Equation S1. Data are presented as mean ± STDV ($n = 4$).

Table S1: Significance values of SNAP loading data (Figure 2B) comparing SNAP-incorporated sponges.

SNAP Loading Significance Values					
**Sponge Porosity** (%)	**66**	**70**	**77**	**83**	**88**
**60**	ns	****	****	****	****
**66**		****	****	****	****
**70**			****	****	****
**77**				****	****
**83**					****

****** ($p < 0.0001$), ns (not significant).**

Table S2: Percent of available SNAP leached from sponges in PBS over 24 h.

SNAP Leached Over Time (%)					
**Sponge Porosity** (%)	**1 h**	**4 h**	**8 h**	**12 h**	**24 h**
**60**	12.9±0.61	31.6±0.58	40.7±2.25	49.4±5.76	75.4±5.13
**66**	16.0±4.43	46.7±8.42	58.8±6.06	70.8±2.69	85.9±2.96
**70**	26.3±2.93	59.4±5.07	75.5±1.07	83.6±1.78	90.0±3.27
**77**	35.5±2.74	70.9±5.61	86.8±3.28	92.5±1.68	94.9±1.73
**83**	51.1±0.66	91.4±1.50	98.2±1.95	99.0±2.02	99.4±1.92
**88**	43.7±4.68	86.2±3.83	97.2±2.54	100±2.40	102±0.09

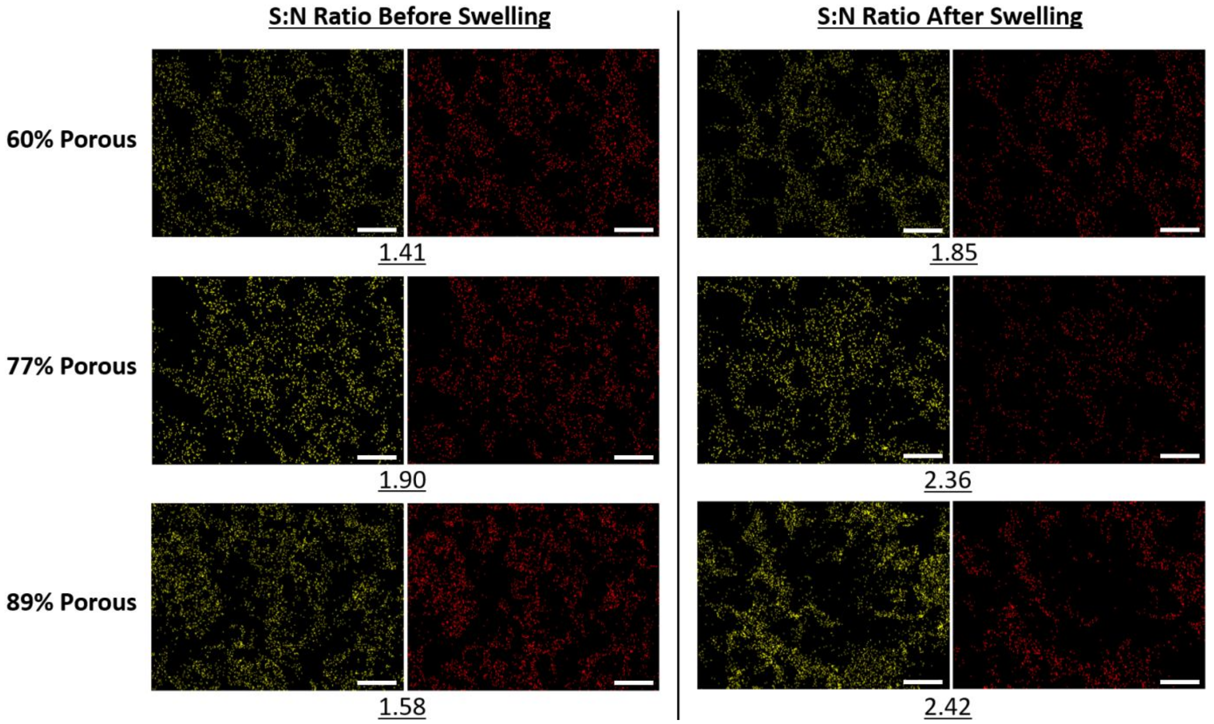

Figure S6. Energy-dispersive X-ray spectroscopy (EDS) analysis of the 60, 77, and 89% porous SNAP-incorporated PDMS sponges before and after submersion in PBS with EDTA at 37 °C for 4 h. Elemental maps show the distribution and intensity of sulfur (S, yellow) and nitrogen (N, red). An increase in the S:N atomic ratio after swelling indicates NO release from SNAP, resulting from cleavage of the S-N bond. The magnitude of the S:N ratio shift was greater in higher-porosity sponges, consistent with enhanced NO release due to increased fluid uptake and SNAP accessibility. Scale bars represent 500 μm.

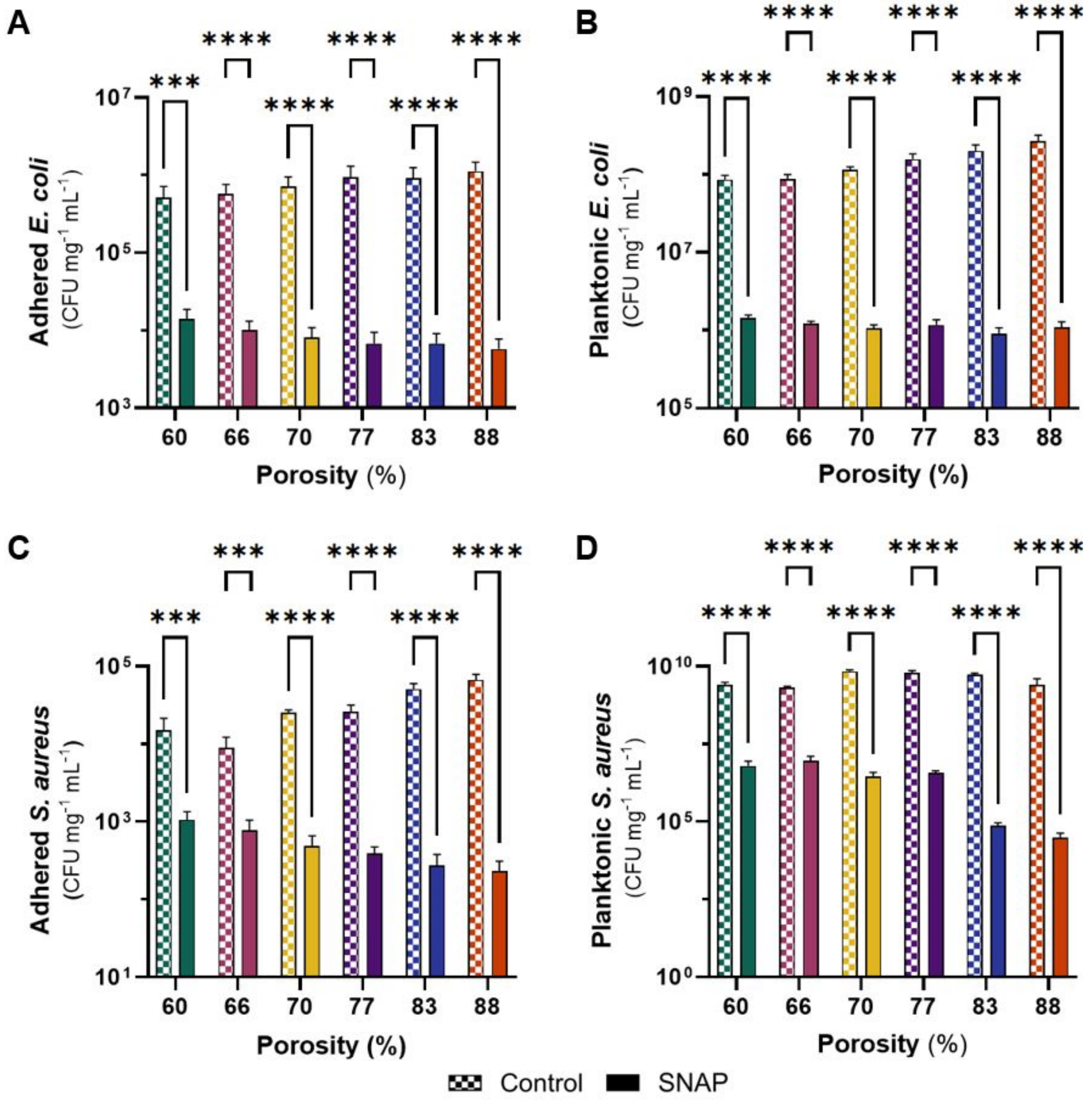

Figure S7. Bacteria CFUs of control and SNAP-incorporated PDMS sponges against adhered **(A)** and planktonic **(B)** *E. coli* and adhered **(C)** and planktonic **(D)** *S. aureus* after 4 h exposure to bacteria. Data are presented as mean of two biological replicates ± SEM (*n = 6*).

Table S3: Significance values of adhered *E. coli* data (Figure 3A) comparing SNAP-incorporated sponges.

Adhered *E. coli* Killing Significance Values

**Sponge Porosity** (%)	**66**	**70**	**77**	**83**	**88**
**60**	***	****	****	****	****
**66**		***	****	****	****
**70**			ns	**	****
**77**				ns	****
**83**					****

****** ($p < 0.0001$), *** ($p < 0.001$), ** ($p < 0.01$), ns (not significant).**

Table S4: Significance values of planktonic *E. coli* data (Figure 3B) comparing SNAP-incorporated sponges.

Planktonic *E. coli* Killing Significance Values

**Sponge Porosity** (%)	**66**	**70**	**77**	**83**	**88**
**60**	ns	****	****	****	****
**66**		****	****	****	****
**70**			ns	****	****
**77**				****	****
**83**					ns

****** ($p < 0.0001$), ns (not significant).**

Table S5: Significance values of adhered *S. aureus* data (Figure 3C) comparing SNAP-incorporated sponges.

Adhered *S. aureus* Killing Significance Values					
**Sponge Porosity** (%)	**66**	**70**	**77**	**83**	**88**
**60**	ns	****	****	****	****
**66**		****	****	****	****
**70**			ns	****	****
**77**				****	****
**83**					ns

****** (p < 0.0001), ns (not significant).**

Table S6: Significance values of planktonic *S. aureus* data (Figure 3D) comparing SNAP-incorporated sponges.

Planktonic *S. aureus* Killing Significance Values					
**Sponge Porosity** (%)	**66**	**70**	**77**	**83**	**88**
**60**	ns	****	***	****	****
**66**		****	*	****	****
**70**			ns	****	****
**77**				****	****
**83**					ns

****** (p < 0.0001), *** (p < 0.001), * (p < 0.05), ns (not significant).**

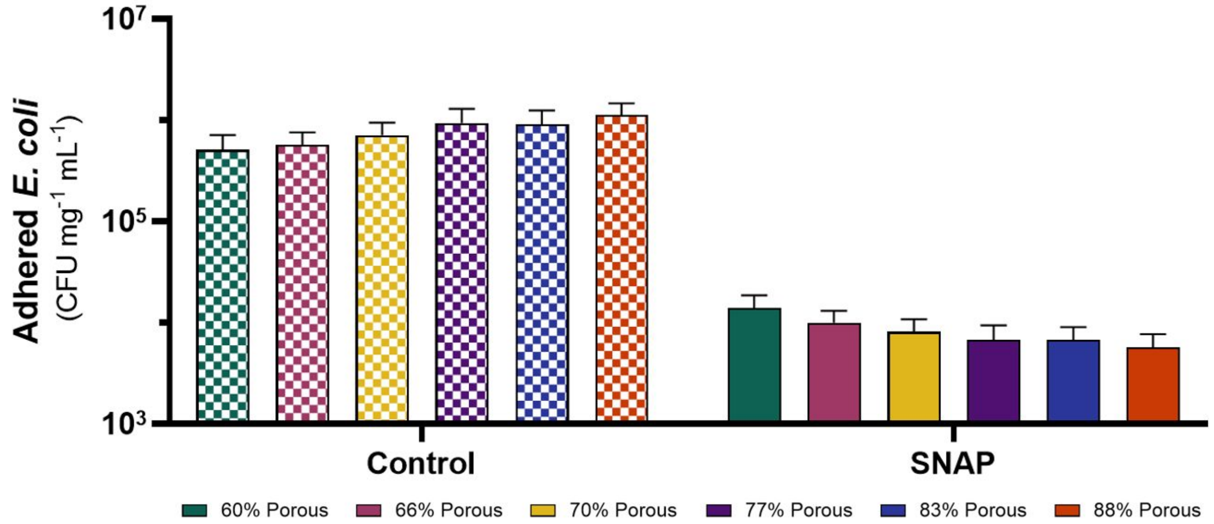

Figure S8. Bacteria CFUs of control and SNAP-incorporated PDMS sponges against adhered *E. coli* after 4 h exposure to bacteria. Data are presented as mean of two biological replicates ± SEM ($n = 6$).

Table S7. Significance values of adhered *E. coli* CFU data comparing sponge types within their sample groups.

	Adhered *E. coli* Control Significance Values					Adhered *E. coli* SNAP Significance Values				
**Sponge Porosity** (%)	**66**	**70**	**77**	**83**	**88**	**66**	**70**	**77**	**83**	**88**
**60**	ns	ns	ns	ns	**	ns	*	**	**	***
**66**		ns	ns	ns	*		ns	ns	ns	ns
**70**			ns	ns	ns			ns	ns	ns
**77**				ns	ns				ns	ns
**83**					ns					ns

***** ($p < 0.001$), ** ($p < 0.01$), * ($p < 0.05$), ns (not significant).**

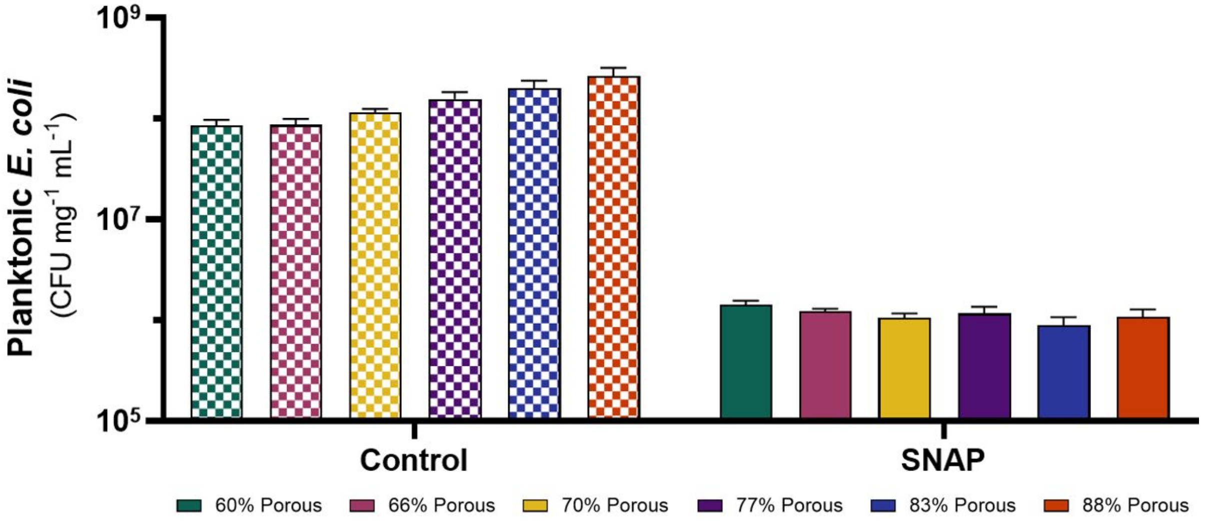

Figure S9. Bacteria CFUs of control and SNAP-incorporated PDMS sponges against planktonic *E. coli* after 4 h exposure to bacteria. Data are presented as mean of two biological replicates ± SEM (*n* = 6).

Table S8. Significance values of planktonic *E. coli* CFU data comparing sponge types within their sample groups.

	**Planktonic *E. coli* Control Significance Values**					**Planktonic *E. coli* SNAP Significance Values**				
**Sponge Porosity** (%)	**66**	**70**	**77**	**83**	**88**	**66**	**70**	**77**	**83**	**88**
**60**	ns	ns	**	****	****	ns	**	ns	****	**
**66**		ns	**	****	****		ns	ns	*	ns
**70**			ns	****	****			ns	ns	ns
**77**				ns	****				ns	ns
**83**					*					ns

****** (p < 0.0001), ** (p < 0.01), * (p < 0.05), ns (not significant).**

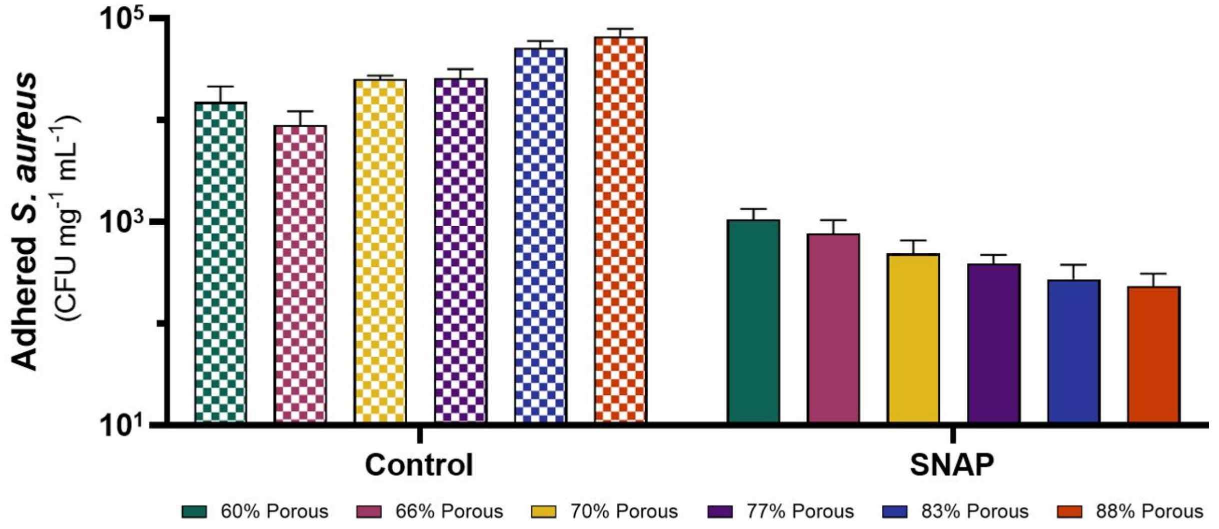

Figure S10. Bacteria CFUs of control and SNAP-incorporated PDMS sponges against adhered *S. aureus* after 4 h exposure to bacteria. Data are presented as mean of two biological replicates ± SEM ($n = 6$).

Table S9. Significance values of adhered *S. aureus* CFU data comparing sponge types within their sample groups.

	Adhered S. *aureus* Control Significance Values					Adhered S. *aureus* SNAP Significance Values				
**Sponge Porosity (%)**	**66**	**70**	**77**	**83**	**88**	**66**	**70**	**77**	**83**	**88**
**60**	ns	ns	ns	****	****	ns	***	****	****	****
**66**		**	**	****	****		ns	*	***	***
**70**			ns	****	****			ns	ns	ns
**77**				****	****				ns	ns
**83**					*					ns

***** ($p < 0.001$), ** ($p < 0.01$), * ($p < 0.05$), ns (not significant).**

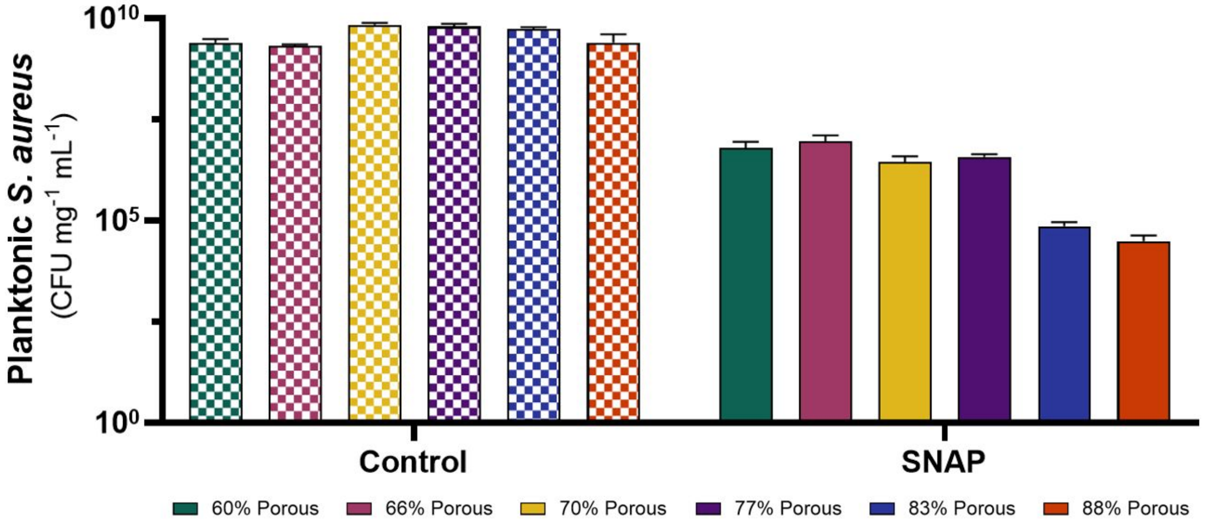

Figure S11. Bacteria CFUs of control and SNAP-incorporated PDMS sponges against planktonic *S. aureus* after 4 h exposure to bacteria. Data are presented as mean of two biological replicates ± SEM (*n* = *6*).

Table S10. Significance values of planktonic *S. aureus* CFU data comparing sponge types within their sample groups.

	**Planktonic S. *aureus* Control Significance Values**					**Planktonic S. *aureus* SNAP Significance Values**				
**Sponge Porosity** (%)	**66**	**70**	**77**	**83**	**88**	**66**	**70**	**77**	**83**	**88**
**60**	ns	****	****	****	ns	ns	*	ns	****	****
**66**		****	****	****	ns		****	***	****	****
**70**			ns	ns	****			ns	ns	ns
**77**				ns	****				*	*
**83**					****					ns

***** (p < 0.001), ** (p < 0.01), * (p < 0.05), ns (not significant).**

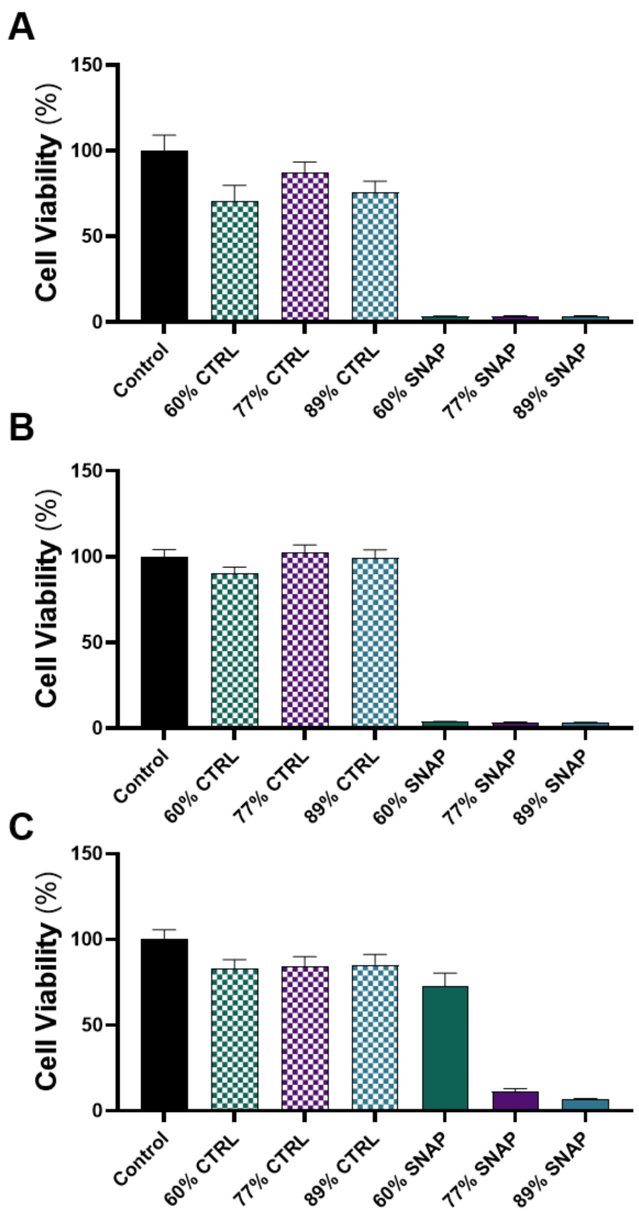

Figure S12. Cytocompatibility assessment of SNAP-incorporated PDMS sponges (60, 77, and 89% porous) via an MTT assay. Leachates were collected after exposing sponges in cell culture media for 4 h and diluted to **(A)** 100, **(B)** 50, and **(C)** 10% concentrations before being applied to L929 mouse fibroblasts for 24 h. Cell viability is expressed as a percentage relative to untreated controls. Data are presented as mean ± SEM (*n = 4*)
